# PGC-1α drives small cell neuroendocrine cancer progression toward an ASCL1-expressing subtype with increased mitochondrial capacity

**DOI:** 10.1073/pnas.2416882121

**Published:** 2024-11-26

**Authors:** Grigor Varuzhanyan, Chia-Chun Chen, Jack Freeland, Tian He, Wendy Tran, Kai Song, Liang Wang, Donghui Cheng, Shili Xu, Gabriella A. Dibernardo, Favour N. Esedebe, Vipul Bhatia, Mingqi Han, Evan R. Abt, Jung Wook Park, Sanaz Memarzadeh, David B. Shackelford, John K. Lee, Thomas G. Graeber, Orian S. Shirihai, Owen N. Witte

**Affiliations:** ^a^Department of Microbiology Immunology and Molecular Genetics, University of California, Los Angeles, CA 90095; ^b^Department of Molecular and Medical Pharmacology, University of California, Los Angeles, CA 90095; ^c^Molecular Biology Interdepartmental Program, University of California, Los Angeles, CA 90095; ^d^Department of Bioengineering, University of California, Los Angeles, CA 90095; ^e^Eli and Edythe Broad Center of Regenerative Medicine and Stem Cell Research, University of California, Los Angeles, CA 90095; ^f^Crump Institute for Molecular Imaging, David Geffen School of Medicine, University of California, Los Angeles, CA 90095; ^g^Jonsson Comprehensive Cancer Center, the David Geffen School of Medicine, University of California, Los Angeles, CA 90095; ^h^Department of Obstetrics and Gynecology, David Geffen School of Medicine, University of California, Los Angeles, CA 90095; ^i^Bioinformatics Interdepartmental Program, University of California, Los Angeles, CA 90095; ^j^Division of Hematology/Oncology, Department of Medicine University of California Los Angeles Jonsson Comprehensive Cancer Center, University of California, Los Angeles, CA 90095; ^k^Human Biology Division, Fred Hutchinson Cancer Center, Seattle, WA 98109; ^l^Department of Pulmonary and Critical Care Medicine, David Geffen School of Medicine, University of California, Los Angeles, CA 90095; ^m^Department of Pathology, Duke University School of Medicine, Durham, NC 27710; ^n^The Veterans Affairs Greater Los Angeles Healthcare System, Los Angeles, CA 90073; ^o^Molecular Biology Institute, University of California, Los Angeles, CA 90095; ^p^California NanoSystems Institute, University of California, Los Angeles, CA 90095; ^q^UCLA Metabolomics Center, University of California, Los Angeles, CA 90095; ^r^University of California Los Angeles Division of Endocrinology, Department of Medicine, David Geffen School of Medicine, University of California, Los Angeles, CA 90095; ^s^Department of Clinical Biochemistry, School of Medicine, Ben Gurion University of The Negev, Beer-Sheva 8410501, Israel; ^t^Parker Institute for Cancer Immunotherapy, University of California, Los Angeles, CA 90095

**Keywords:** PGC-1a, ASCL1, oxidative phosphorylation, lung cancer, prostate cancer

## Abstract

Adenocarcinomas from multiple tissues can converge to treatment-resistant small cell neuroendocrine (SCN) cancers composed of ASCL1, POU2F3, NEUROD1, and YAP1 subtypes. We investigated how mitochondrial metabolism influences SCN cancer (SCNC) progression. Extensive bioinformatics analyses encompassing thousands of patient tumors and human cancer cell lines uncovered enhanced expression of proliferator-activatedreceptor gamma coactivator 1-alpha (PGC-1α), a potent regulator of mitochondrial oxidative phosphorylation (OXPHOS), across several SCNCs. PGC-1α correlated tightly with increased expression of the lineage marker Achaete-scute homolog 1, (ASCL1) through a positive feedback mechanism. Analyses using a human prostate tissue-based SCN transformation system showed that the ASCL1 subtype has heightened PGC-1α expression and OXPHOS activity. PGC-1α inhibition diminished OXPHOS, reduced SCNC cell proliferation, and blocked SCN prostate tumor formation. Conversely, PGC-1α overexpression enhanced OXPHOS, validated by small-animal Positron Emission Tomography mitochondrial imaging, tripled the SCN prostate tumor formation rate, and promoted commitment to the ASCL1 lineage. These results establish PGC-1α as a driver of SCNC progression and subtype determination, highlighting metabolic vulnerabilities in SCNCs across different tissues.

## Significance

Our study provides functional evidence that metabolic reprogramming can directly impact cancer phenotypes and establishes proliferator-activated receptor gamma coactivator 1-alpha (PGC-1α)-induced mitochondrial metabolism as a driver of small cell neuroendocrine cancer (SCNC) progression and lineage determination. These mechanistic insights reveal common metabolic vulnerabilities across SCNCs originating from multiple tissues, opening additional avenues for pan-SCN cancer therapeutic strategies.

Epithelial cancers from various tissues, including the prostate, lung, and bladder, can converge to an aggressive small cell neuroendocrine (SCN) phenotype under targeted treatments (1–3). These phenotypically similar, small cell neuroendocrine cancers (SCNCs) are classified as high-grade, poorly differentiated small cell carcinomas that commonly express neuroendocrine markers like chromogranin A (CHGA), neural cell adhesion molecule 1 (NCAM1), and synaptophysin (SYP) (4). With a dismal median survival rate between 7 to 16 mo (5), there is a pressing need for a better mechanistic understanding of disease progression to inform new therapeutic strategies.

SCNCs are composed of multiple subtypes defined by prominent expression of lineage markers. This is best exemplified in SCLC, which is categorized into ASCL1, NEUROD1, POU2F3, and YAP1 subtypes (6, 7). Recent studies have identified corresponding subtypes in SCN prostate cancer, including ASCL1, POU2F3/ASCL2, and NEUROD1 (8–10). A growing body of research across numerous SCNC models has highlighted the plastic nature of differentiation toward and between these subtypes (6, 7, 11–13).

Complementing the molecular heterogeneity within SCNCs, recent studies have begun to highlight the metabolic diversity across different SCLC subtypes (14–18). A correlation has been observed between ASCL1 expression levels and a dependence on inosine monophosphate dehydrogenase, a key enzyme in guanosine biosynthesis (14). Thus, metabolic reprogramming might regulate lineage plasticity in SCNCs.

A key aspect of metabolic reprogramming in cancer is the shift toward aerobic glycolysis (Warburg effect), characterized by increased glucose consumption and lactate secretion, even in the presence of ampleoxygen (19). This seminal discovery led to a longstanding belief that cancer cells have dysfunctional mitochondria. However, recent studies have countered this perspective, showing that many cancers actually have functional mitochondria fully capable of oxidative phosphorylation (OXPHOS) (20). In fact, some cancers have a heightened reliance on OXPHOS (21), which is sometimes linked with therapy resistance (22–26). This emerging understanding has sparked significant interest in OXPHOS inhibitors as promising candidates for cancer therapy (27–29). However, their utility in treating SCNCs remains unclear owing to an incomplete understanding of the metabolic dynamics in these aggressive cancers. Although increased aerobic glycolysis has been observed in SCN prostate cancer (30), the OXPHOS status in SCNC subtypes remains unclear. Therefore, we reasoned that exploring mitochondrial function in SCNCs may reveal pivotal insights into their pathophysiology.

To explore the influence of mitochondrial function on SCNC development, we focused on peroxisome proliferator-activated receptor gamma coactivator 1-alpha (PGC-1α) (31), a transcriptional coactivator widely recognized as a potent regulator of mitochondrial biogenesis and OXPHOS. Originally identified for its role in cold-induced adaptive thermogenesis through the regulation of OXPHOS genes (32), PGC-1α has since been implicated in metabolic reprogramming during cancer development, demonstrating robust but varied effects across cancer types (33). In prostate cancer, it has been found to support the growth of localized, androgen-dependent tumors (34, 35) while restraining the progression of androgen-independent, metastatic forms (36–38). In melanoma, PGC-1α is associated with tumors that exhibit enhanced mitochondrial function (39) and a reduced metastatic potential (40). It suppresses metastasis in non–small cell lung cancer (NSCLC) (41), but promotes metastasis in other cancer models (42). The role of PGC-1α in SCNCs remains unknown, and thus unraveling its specific effects could provide key insights into potential therapeutic targets or prognostic factors.

To investigate the role of PGC-1α in regulating SCNC development, we performed an integrated computational and functional investigation. Bioinformatics analyses of 10,529 patient tumors and 1,466 human cancer cell lines were performed, followed by functional and mechanistic analyses using multiple model systems including a human prostate tissue-derived SCN transformation system (2, 3, 10). This system enables the transformation of normal human lung epithelial cells, prostate epithelial cells, or bladder urothelial cells into SCNC via transduction with an oncogene cocktail abbreviated as PARCB (TP53DN, myr-AKT, shRB1, c-MYC, and BCL2). This PARCB model generates two distinct tumor lineages characterized by either stem-like (POU2F3/ASCL2 subtype) or neuroendocrine features (ASCL1 subtype) that closely resemble the POU2F3 and ASCL1 subtypes seen in clinical SCNCs (10). Here, we reveal the metabolic heterogeneity among SCNC subtypes, defined by PGC-1α and OXPHOS levels. We also identify PGC-1α as a driver in the ASCL1 lineage and uncover OXPHOS as a broad metabolic vulnerability across SCNCs originating from multiple tissues.

## Results

### Elevated PGC-1α in Clinical SCNCs Correlates with Increased ASCL1 Expression and Neuroendocrine Differentiation.

**Fig. 1.**
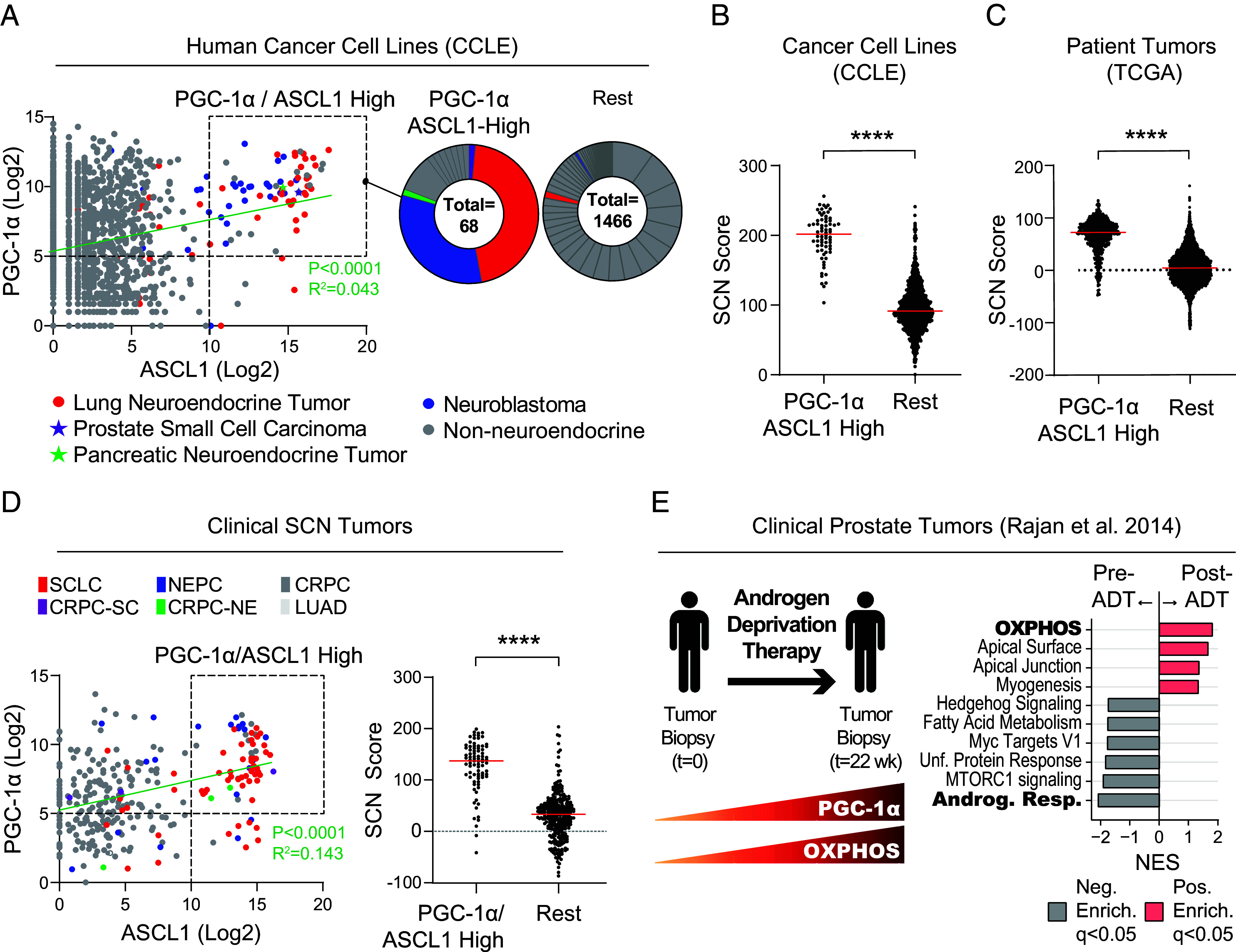
Elevated PGC-1α in clinical SCNC correlates with increased ASCL1 expression and neuroendocrine differentiation. (*A*) Gene expression analysis across all human cancer cell lines from the CCLE. (*Left*) Coexpression analysis between PGC-1α and ASCL1, highlighting samples with high expression levels of both genes (boxed region). (*Right*) Pie charts illustrating the proportion of cancer types exhibiting elevated coexpression of PGC-1α and ASCL1, in contrast to the rest of the samples. Related to *SI Appendix*, Fig. S1*A*. (*B*) Transcriptomic examination of SCN differentiation in CCLE human cancer cell lines. SCN scores [defined previously (3) and detailed in the *Materials and Methods*] are calculated for cells showing heightened PGC-1α and ASCL1 expression versus the rest of the cells. Related to Fig. 1*A* and *SI Appendix*, Fig. S1*B*. (*C*) A transcriptomic analysis of SCN differentiation in all the patient tumors from the TCGA database shown in *SI Appendix*, Fig. S1*C*. SCN scores are calculated from the boxed tumor samples with elevated PGC-1α and ASCL1 expression compared with the rest of the samples. Related to *SI Appendix*, Fig. S1*C*. (*D*) Gene expression analysis in multiple clinical SCNC datasets. (*Left*) Coexpression analysis of PGC-1α and ASCL1. (*Right*) SCN scores of the boxed samples with elevated PGC-1α and ASCL1 expression compared with the rest of the samples. (*E*) Transcriptomic analyses in clinical prostate cancer tumors before and after ADT with enzalutamide. (*Left*) A schematic overview. (*Right*) Gene set enrichment analysis (GSEA) of differentially expressed genes in pre- versus post ADT-treated tumors. See also *SI Appendix*, Fig. S1*F*. Related to *SI Appendix*, Fig. S1. See *SI Appendix* for statistical analyses and datasets used.

To investigate the role of mitochondrial metabolism in SCNCs, we conducted bioinformatics analyses in a wide range of human cancer cell lines, human primary tumors, and clinical SCN datasets. We conducted a coexpression analysis of PGC-1α against the four SCNC lineage markers—ASCL1, POU2F3, NEUROD1, and YAP1—across all 1,466 human cancer cell lines from the Cancer Cell Line Encyclopedia (CCLE). While PGC-1α does not correlate with POU2F3, NEUROD1, or YAP1 (*SI Appendix*, Fig. S1*A*), it shows positive correlation with the lineage marker ASCL1, a pioneer transcription factor that is essential for neuronal commitment and SCNC progression (43, 44) (Fig. 1*A*). Notably, we observed a distinct subpopulation of cell lines with high ASCL1 expression that also have high PGC-1α expression “PGC-1α/ASCL1-High.” (Fig. 1 *A*, *Left*). To determine the cancer type composition of the PGC-1α/ASCL1-High subpopulation compared to the rest of the samples, we quantified the percentage of cell lines with SCN features in both groups. The PGC-1α/ASCL1-High subpopulation is predominantly composed of SCNCs (54/68, 79%) (Fig. 1 *A*, *Right*), whereas only a small fraction of the rest of the cells (88/1,466, 6%) are SCNCs. Consistently, PGC-1α expression is elevated in SCLC and neuroblastoma cell lines compared with NSCLC (*SI Appendix*, Fig. S1*B*). These observations suggest that PGC-1α and ASCL1 are co-upregulated in SCNCs from multiple tissues.

To determine whether PGC-1α/ASCL1-High cells show increased SCN differentiation, we applied a well-established SCN score derived from a pan-cancer transcriptomic analysis (1). PGC-1α/ASCL1-High cells from the CCLE show a marked increase in SCN score compared to the rest of the samples, indicating heightened SCN differentiation. (Fig. 1*B*). A consistent result was seen across 10,529 solid tumors from The Cancer Genome Atlas (TCGA) (Fig. 1*C* and *SI Appendix*, Fig. S1*C*) and in clinical SCN samples from various cancers, including lung adenocarcinoma (LUAD), castration-resistant prostate cancer (CRPC), neuroendocrine prostate cancer (NEPC), and SCLC (Fig. 1*D*). Consistent with these observations, PGC-1α expression is increased in SCN prostate cancer compared with CRPC and in SCLC compared with NSCLC (*SI Appendix*, Fig. S1*D*). Of note, the increased PGC-1α expression in SCLC is largely attributable to the ASCL1 subtype (*SI Appendix*, Fig. S1 *D*, *Right*). A positive correlation between PGC-1α and ASCL1 was also observed in high-grade serous ovarian carcinomas (HGSOC), which share genomic alterations with neuroendocrine tumors including mutations in TP53, loss of RB1, and amplification of MYC (45). Thus PGC-1α and ASCL1 are co-upregulated in multiple clinical tumor types with heightened SCN differentiation.

Given the known contribution of androgen deprivation therapy (ADT) to SCN prostate cancer progression (46, 47), we next examined its on PGC-1α expression in clinical prostate cancer (Fig. 1*E*).

ADT-treated tumors show increased PGC-1α expression, which correlated positively with ASCL1 (*SI Appendix*, Fig. S1*F*). Further, Gene set enrichment analysis (GSEA) revealed that OXPHOS is a top enriched pathway in post-ADT tumors (Fig. 1*E*). Collectively, these data indicate that PGC-1α expression is upregulated in multiple SCNCs and uncover a previously undescribed link between PGC-1α, ASCL1, and SCN differentiation.

### Enhanced PGC-1α Expression and OXPHOS Activity in SCN Prostate Cancer within the ASCL1 Tumor Subtype.

**Fig. 2.**
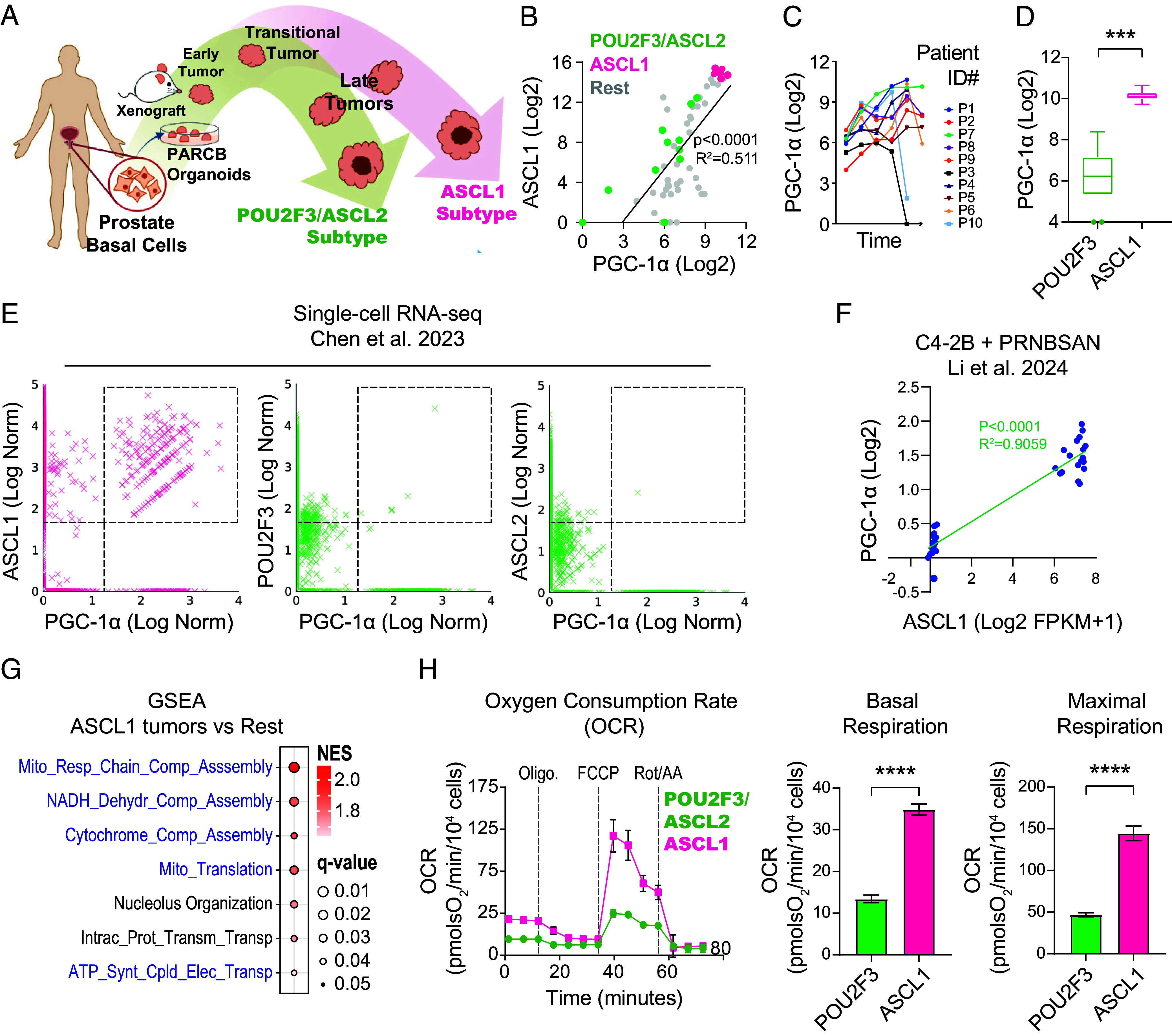
Enhanced PGC-1α expression and OXPHOS activity in SCN prostate cancer within the ASCL1 tumor subtype. (*A*) Schematic illustrating PARCB-induced SCN prostate transformation, highlighting the bifurcation of end-stage tumors into the POU2F3/ASCL2 and ASCL1 lineages. The schematic is modified from Chen et al. (10). (*B*) Coexpression analysis of PGC-1α and ASCL1 during PARCB prostate transformation. (*C*) Analysis of PGC-1α expression levels during PARCB transformation in all 10 patient series showcasing increased PGC-1α expression over time. The final time points of patient series #3 (P3) and #10 (P10) correspond to the development of the POU2F3/ASCL2 subtype. (*D*) Expression levels of PGC-1α in POU2F3/ASCL2 versus ASCL1 PARCB tumor subtypes. (*E*) Single cell gene expression levels of PGC-1α versus ASCL1, POU2F3, and ASCL2 from the PARCB time course tumors (10). (*F*) Coexpression analysis of PGC-1α and ASCL1 in C4-2B cells from the PRNBSAN transformation system. Log2 values are Log2 FPKM+1. (*G*) GSEA in the ASCL1 tumor subtype versus the rest of the PARCB time course samples. Gene Ontology Biological Process (GOBP) gene sets were used. (*H*) Seahorse respirometry in PARCB tumor-derived cell lines from both the POU2F3/ASCL2 and ASCL1 subtypes. See *SI Appendix* for inhibitor details. Related to *SI Appendix*, Figs. S2–S7. See *SI Appendix* for statistical analyses and datasets used.

Having established a strong correlation between PGC-1α and ASCL1 in multiple clinical SCNCs, we next sought to identify appropriate model systems for studying the functional role of PGC-1α in SCNC progression and lineage determination. We utilized a human tissue-derived PARCB-induced SCN transformation model (10), which produces ASCL1 and POU2F3/ASCL2 subtypes, closely resembling clinical SCNC subtypes (Fig. 2*A*). Coexpression analysis between PGC-1α and ASCL1 across all PARCB time course samples revealed a tight, positive correlation, with the highest expression of both genes found exclusively in the ASCL1 subtype (Fig. 2*B*). Further, PGC-1α expression markedly increased in the majority of tumor series derived from distinct donor tissue series, with a sudden drop in PGC-1α expression levels in two patient series (P3 and P10) upon development of the POU2F3/ASCL2 subtype (Fig. 2*C*). Consistently, ASCL1 subtype tumors demonstrate a 16-fold higher expression of PGC-1α compared with the POU2F3/ASCL2 subtype (Fig. 2*D*). Further, single-cell RNA-sequencing (scRNA-seq) analysis revealed that high PGC-1α expression was almost exclusively associated with ASCL1 (Fig. 2*E*). Thus, the PARCB model recapitulates the analyses in clinical tumors described in Fig. 1, making it suitable for downstream functional experiments.

To determine whether transcription factors (TFs) coactivated by PGC-1α are more accessible in the ASCL1 subtype than in the POU2F3/ASCL2 subtype, Hypergeometric Optimization of Motif EnRichment (HOMER) analysis was performed to compare the accessible peaks in both tumor subtypes. In the ASCL1 tumor subtype, there was a notable enrichment of transcription factors coactivated by PGC-1α, including HNF4A and PPPARA, which were among the top 10 most significantly enriched (*SI Appendix*, Fig. S2 *A* and *B*). HNF4A, recently demonstrated to define a fifth SCNC subtype (48), demonstrates a strong and positive correlation with PGC-1α in PARCB tumors (*SI Appendix*, Fig. S2*C*). Consistently, HNF4A shows a positive correlation with PGC-1α across multiple clinical SCNC datasets (*SI Appendix*, Fig. S2*D*). To explore HNF4A’s correlation with PGC-1α and ASCL1, we analyzed the coexpression of all three genes (*SI Appendix*, Fig. S3 *A*–*D*). Most ASCL1-high samples exhibit low HNF4A expression. However, a subset of PGC-1α- and ASCL1-high samples also exhibit high HNF4A expression.

Given the shared molecular alterations driven by a common set of oncogenes in both ASCL1 and POU2F3/ASCL2 subtypes, we investigated whether ASCL1, a pioneer transcriptional activator capable of binding to and opening compacted chromatin, could directly interact with PGC-1α to enhance its expression specifically in the ASCL1 subtype. We analyzed ASCL1 ChIP-Seq datasets from SCN lung and prostate cancer cell lines, as well as in LuCaP prostate cancer patient-derived xenografts (PDXs) (*SI Appendix*, Figs. S4–S6). While ASCL1 showed some binding to PGC-1α, inconsistent binding patterns across datasets and models hinder definitive conclusions about ASCL1’s direct regulation of PGC-1α.

Next, we sought to determine whether a functional connection exists that does not solely rely on direct DNA interaction. To this end, we investigated whether exogenous ASCL1 expression could upregulate PGC-1α. Indeed, ASCL1 overexpression in two PARCB POU2F3/ASCL2 tumor-derived cell lines led to increased PGC-1α expression in both cell lines (10) (*SI Appendix*, Fig. S7*A*). To ensure that our findings extend beyond PARCB cell lines, we utilized an in vitro model for SCN prostate cancer differentiation using the androgen-insensitive C4-2B cell line. This model induces SCN differentiation by incorporating a specific set of SCN oncogenes and transcription factors, namely PRNBSAN (dominant-negative TP53, shRB1, MYCN, BCL2, SRRM4 combined with ASCL1, or NEUROD1) (*SI Appendix*, Fig. S7*B*). Consistent with findings in the PARCB cell lines, we observed a strong functional connection between PGC-1α and ASCL1 expression, evidenced by an R^2^ value of 0.9059 (Fig. 2*F*). Moreover, PGC-1α levels also correlated with enhanced SCN differentiation in this model (*SI Appendix*, Fig. S7*C*). Collectively, these findings across multiple model systems establish ASCL1 as a positive regulator of PGC-1α expression.

We next defined global transcriptional changes associated with the development of the ASCL1 subtype. GSEA was performed in tumors of the ASCL1 subtype versus all the other stages of PARCB transformation (Fig. 2*G*). ASCL1 tumors had pronounced upregulation of OXPHOS genes (Fig. 2*G*). To assess whether increased OXPHOS gene expression led to higher activity, we conducted Seahorse respirometry, which provided a functional profile of OXPHOS. This analysis revealed that PARCB-ASCL1 tumor-derived cell lines showed significantly higher basal and maximal oxygen consumption rates compared to the POU2F3/ASCL2 subtype (Fig. 2*H*), indicating increased OXPHOS activity. Furthermore, the ASCL1 tumor subtype had higher expression of both subunits of the mitochondrial pyruvate carrier (*SI Appendix*, Fig. S7*D*), which allows entry of pyruvate into the mitochondrion to fuel OXPHOS. Collectively, these findings demonstrate that the ASCL1 subtype exhibits enhanced PGC-1α expression and OXPHOS activity.

### PGC-1α Inhibition Blunts OXPHOS, Reduces the Growth of SCNC Cell Lines, and Blocks SCN Prostate Tumor Formation.

To determine whether PGC-1α is functionally important for PARCB tumor progression, PGC-1α was inhibited during PARCB prostate transformation using shRNA interference (*SI Appendix*, Fig. S8*A*). PGC-1α inhibition was verified using Western blotting in 293Ts (*SI Appendix*, Fig. S8*B*) and using RT-qPCR in PARCB organoids (*SI Appendix*, Fig. S8*C*) before xenografting into immunocompromised mice for tumor formation. PGC-1α inhibition blocked PARCB tumor growth, underscoring its requirement for PARCB prostate tumor initiation.

**Fig. 3.**
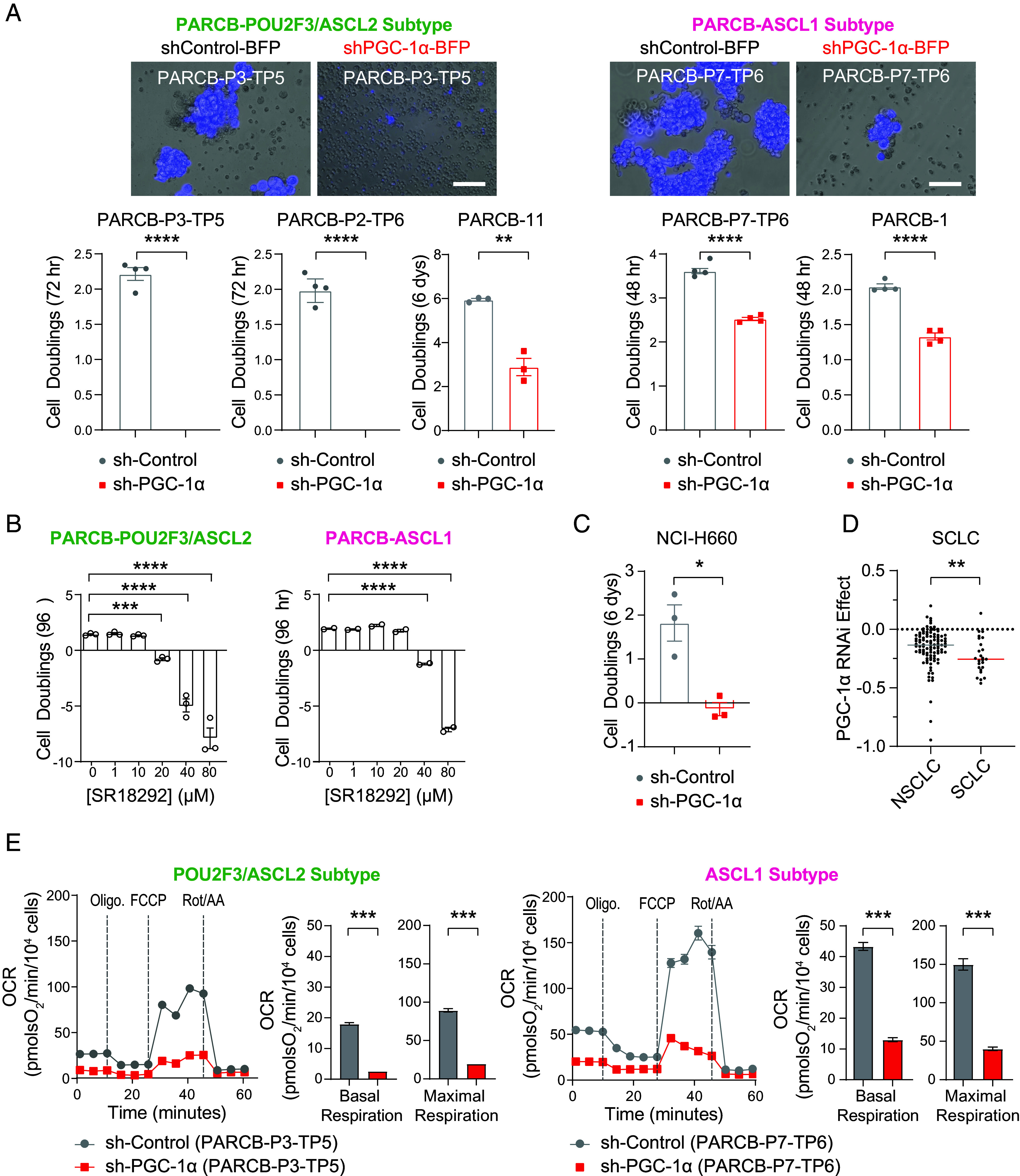
PGC-1α inhibition blunts OXPHOS, reduces the growth of SCNC cell lines, and blocks SCN prostate tumor formation. (*A*) Cell proliferation analysis in PARCB tumor-derived cell lines comparing PGC-1α knockdown cells to control. (*B*) Cell proliferation analysis of PARCB tumor-derived cell lines treated with SR-18292, a pharmacological inhibitor of PGC-1α. (*C*) Cell proliferation analysis of a patient-derived SCN prostate cancer cell line (NCI-H660) with PGC-1α knockdown compared with control. (*D*) PGC-1α inhibition (RNAi) effects on cell proliferation, represented by dependency scores from the DepMap database. See *SI Appendix* for details. (*E*) Seahorse respirometry in PARCB cell lines derived from the POU2F3/ASCL2 and ASCL1 tumor subtypes. See *SI Appendix* for inhibitor details. Related to *SI Appendix*, Fig. S8. See *SI Appendix* for statistical analyses and datasets used.

Since PARCB tumors could not form under PGC-1α inhibition, we explored its role in the proliferation of PARCB tumor–derived prostate cancer cell lines. PGC-1α inhibition using shRNA and the small molecule SR-18292 (49), resulted in reduced proliferation in both ASCL1 and POU2F3/ASCL2 subtypes, with a stronger effect observed in the POU2F3/ASCL2 subtype (Fig. 3 *A* and *B*).

To determine whether this effect of PGC-1α inhibition extends beyond the PARCB-derived cell lines, we first examined its effect on PRNBSA-induced SCN prostate cancer progression (model described in *SI Appendix*, Fig. S7*B*). PGC-1α inhibition blocked the PRNBSA-mediated growth of both C4-2B and LNCaP cell lines (*SI Appendix*, Fig. S8 *D* and *E*).

Next, we tested the effect of PGC-1α inhibition on patient-derived SCN prostate and lung cancer cell lines. PGC-1α inhibition reduced the growth of the sole patient-derived SCN prostate cancer cell line, NCI-H660 (Fig. 3*C*). Similarly, analysis of PGC-1α inhibition in the Cancer Dependency Map (DepMap) revealed a stronger anti-proliferative effect in SCLC compared with NSCLC (Fig. 3*D*). Taken together, these findings indicate that PGC-1α inhibition blunts the growth of both SCN prostate and lung cancer cell lines derived from multiple model systems and patient donors.

To understand the mechanism behind PGC-1α inhibition’s impact on SCNC development we examined whether PGC-1α inhibition would blunt OXPHOS in SCN prostate cancer cell lines. Accordingly, we inhibited PGC-1α in ASCL1 and POU2F3/ASCL2 cell lines using shRNA and performed Seahorse respirometry to examine the effect on mitochondrial function. PGC-1α inhibition downregulated basal and maximal respiration in both the ASCL1 and POU2F3/ASCL2 subtypes (Fig. 3*E*). These findings collectively suggest that PGC-1α regulates the initiation of PARCB prostate tumors and maintains proliferation of cultured SCNC cell lines by sustaining OXPHOS.

### OXPHOS Inhibition Blunts SCN Prostate and Lung Cancer Cell Line Proliferation.

**Fig. 4.**
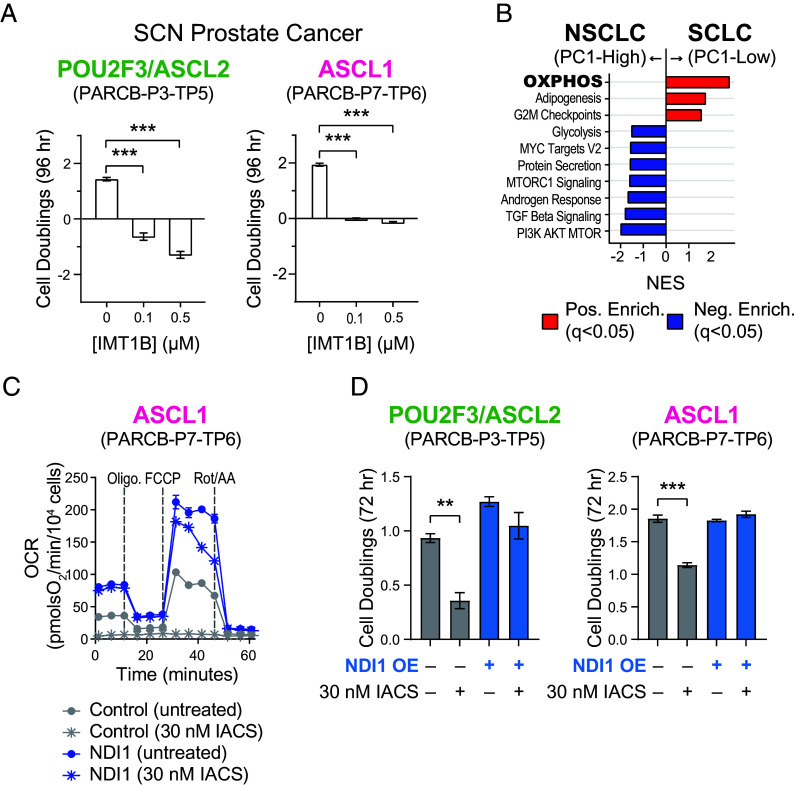
OXPHOS inhibition blunts SCN prostate and lung cancer cell line proliferation. (*A*) Cell proliferation analysis of cell lines derived from the SCN prostate POU2F3/ASCL2 and ASCL1 tumor subtypes treated with IMT1B, a mitochondrial DNA-directed RNA polymerase (POLRMT) inhibitor to block OXPHOS. (*B*) Analysis of differentially dependent genes in NSCLC and SCLC cell lines. Normalized enrichment scores of gene sets that separate PC1 low (SCLC) and PC1 high (NSCLC). (*C*) Seahorse respirometry in cell lines derived from the PARCB ASCL1 tumor subtype with the indicated conditions. See *SI Appendix* for inhibitor details. See *SI Appendix*, Figure S9C for POU2F3 data. (*D*) Cell proliferation analysis of cell lines derived from the SCN prostate cancer POU2F3/ASCL2 and ASCL1 subtypes with the indicated treatments.

To further corroborate OXPHOS as PGC-1α’s mechanism of action, we tested whether OXPHOS specifically is required for the proliferation of SCNC cell lines. First, PARCB cell lines derived from the ASCL1 and POU2F3/ASCL2 tumor subtypes were treated with IMT1B, a small molecule inhibitor of mitochondrial RNA polymerase (POLRMT), which blocks mitochondrial DNA transcription thereby downregulating all of the mitochondrially encoded respiratory chain complexes (50). IMT1B treatment blunted cell proliferation in both SCN prostate and lung cancer cell lines with approximately twice the potency in the POU2F3/ASCL2 subtype (Fig. 4*A* and *SI Appendix*, Fig. S9*A*).

We next examined the effect of OXPHOS gene deletion in SCN lung cancer by investigating genetic dependencies of 104 lung cancer cell lines in the DepMap database. Unsupervised principal component analysis (PCA) using the DepMap dependency scores revealed a distinct separation between SCLC and NSCLC samples, indicating unique genetic dependencies between the two groups (*SI Appendix*, Fig. S9*B*). Pathway enrichment analysis from the top and bottom 100 PC1 loading genes indicated that OXPHOS is a top enriched pathway in SCLC versus NSCLC cell lines, implying that OXPHOS gene deletion has a stronger antiproliferation effect in SCLC versus NSCLC (Fig. 4*B*).

Treatment with a clinical-grade respiratory chain complex I inhibitor, IACS-010759 abolished OXPHOS in cell lines derived from both subtypes (Fig. 4*C* and *SI Appendix*, Fig. S9*C*). To confirm the inhibitor’s specificity and ensure that the observed effects were directly due to complex I inhibition, NDI1, a yeast complex I (28, 51) was overexpressed concurrently with complex I inhibition. NDI1 overexpressed fully rescued the OXPHOS inhibition caused by IACS-010759 (Fig. 4*C* and *SI Appendix*, Fig. S9*C*). Interestingly, NDI1 overexpression boosted the oxygen consumption rate (OCR) above baseline levels only in the ASCL1 subtype (Fig. 4*C* and *SI Appendix*, Fig. S9*C*).

IACS-010759 treatment reduced proliferation of cell lines derived from both subtypes, with a more pronounced effect in the POU2F3/ASCL2 subtype (Fig. 4*D*). These antiproliferation effects caused by IACS-010759 were fully rescued by NDI1 overexpression (Fig. 4*D*), indicating high specificity of the inhibitor. A similar effect was observed in SCN lung cancer cell lines, with a more pronounced effect in the POU2F3 subtype (*SI Appendix*, Fig. S9*D*). Collectively, these results indicate that PGC-1α and OXPHOS inhibition are metabolic vulnerabilities in SCN cancers of both the prostate and lung.

### PGC-1α Promotes OXPHOS in the Prostate Cancer ASCL1 Subtype.

**Fig. 5.**
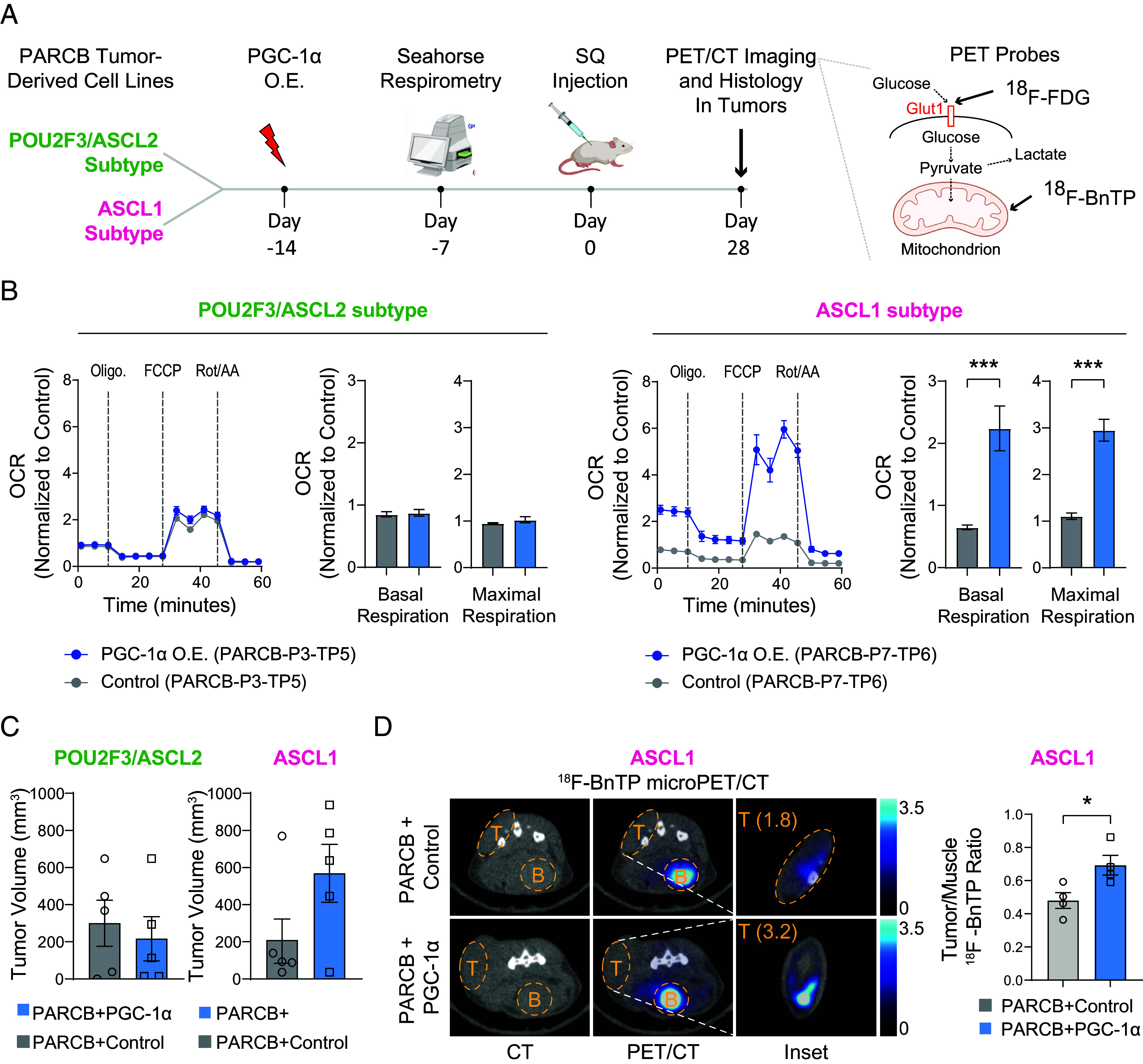
PGC-1α overexpression promotes OXPHOS in the SCN prostate cancer ASCL1 subtype. (*A*, *Left*) Overview of experimental strategy. (*Right*) Details on the PET probes utilized and their mechanisms of action. (*B*) Seahorse respirometry in POU2F3/ASCL2 and ASCL1 PARCB tumor-derived cell lines. See *SI Appendix* for inhibitor details. (*C*) Caliper measurements of tumors initiated by subcutaneous injection of POU2F3/ASCL2 and ASCL1 PARCB cell lines with PGC-1α overexpression versus control. (*D*, *Left*) Representative ^18^F-FBnTP transverse PET-CT images of mice with subcutaneous tumor implantation. Uptake of PET probe was measured as the maximum percentage of injected dose per cubic centimeter (ID%/cc). Tumors are labeled “T,” and bladders are labeled “B.” The *Inset* has been contrast enhanced to show the relatively low uptake of this probe in subcutaneous tumors, as reported previously (52). (*Right*) quantification of ^18^F-BnTP uptake in the indicated groups. Values are normalized to PET signal from adjacent skeletal muscle. Related to *SI Appendix*, Figs. S10 and S11. See *SI Appendix* for statistical analyses and datasets used.

Because PGC-1α inhibition reduced OXPHOS activity in SCNC cell lines, we wondered whether overexpressing PGC-1α would be sufficient to promote OXPHOS and induce lineage plasticity. PGC-1α overexpression increased both PGC-1α and OXPHOS protein levels in a panel of PARCB cell lines developed previously (2) (*SI Appendix*, Fig. S10*A*). Next, PGC-1α was overexpressed specifically in POU2F3/ASCL2 and ASCL1 PARCB tumor-derived cell lines, and its effects on OXPHOS activity and neuroendocrine differentiation were measured. This analysis utilized Seahorse respirometry, followed by in vivo micropositron emission tomography (PET) and computed tomography (microPET/CT) imaging, and histological analyses (Fig. 5*A*). PGC-1α overexpression upregulated OXPHOS activity in cell lines derived from the ASCL1 but not POU2F3/ASCL2 subtype (Fig. 5*B*). This result is consistent with the ASCL1 subtype’s selective OXPHOS enhancement from yeast Complex I overexpression seen in Fig. 4*C*, suggesting a relatively flexible metabolic state in the ASCL1 subtype.

To check whether the increased OXPHOS caused by PGC-1α overexpression in cell lines would translate to higher bioenergetics in tumors, ASCL1, or POU2F3/ASCL2 cell lines with and without PGC-1α overexpression were xenografted into immunocompromised mice for tumor initiation and subsequent microPET/CT imaging. PGC-1α overexpression promoted tumor growth only in the ASCL1 subtype, with responses varying from a twofold to fivefold increase in tumor volume (Fig. 5*C*). To evaluate the metabolic changes induced by PGC-1α overexpression in these tumors, we employed two distinct PET probes for microPET/CT imaging, ^18^F-FDG and ^18^F-BnTP (Fig. 5*A* and *SI Appendix*, Fig. S10 *B* and *C*). ^18^F-FDG, a glucose analog, is extensively used in clinical PET imaging to measure cellular glucose uptake. While its accumulation in tumors indicates increased glucose uptake, it does not provide information on the specific metabolic pathways, such as whether glucose gets incorporated into the TCA cycle to fuel OXPHOS. To address this limitation, we also utilized ^18^F-BnTP, a lipophilic cation designed to accumulate specifically in the mitochondrial inner membrane and measure mitochondrial membrane potential (52). This accumulation labels mitochondria with intact OXPHOS activity, offering a direct measure of mitochondrial activity and content in tumors in vivo. PGC-1α overexpression in the ASCL1 subtype enhanced uptake of both ^18^F-BnTP (Fig. 5*D*) and ^18^F-FDG (*SI Appendix*, Fig. S10*D*). These effects were not seen in the POU2F3/ASCL2 subtype (*SI Appendix*, Fig. S10*E*). Histological analysis from tumors derived from both subtypes showed no obvious changes in SCN differentiation upon PGC-1α overexpression in either subtype (*SI Appendix*, Fig. S11).

Collectively, the data indicate that, relative to the POU2F3/ASCL2 subtype, the ASCL1 subtype demonstrates high metabolic plasticity, exhibiting a marked OXPHOS response to PGC-1α and NDI1 overexpression. Moreover, the data indicate that despite the ASCL1 subtype’s greater OXPHOS response to PGC-1α overexpression, PGC-1α alone is not sufficient to trigger additional neuroendocrine differentiation in these terminally differentiated cell lines.

### PGC-1α Upregulates OXPHOS and Drives SCN Prostate Cancer Toward an ASCL1-Expressing Lineage.

**Fig. 6.**
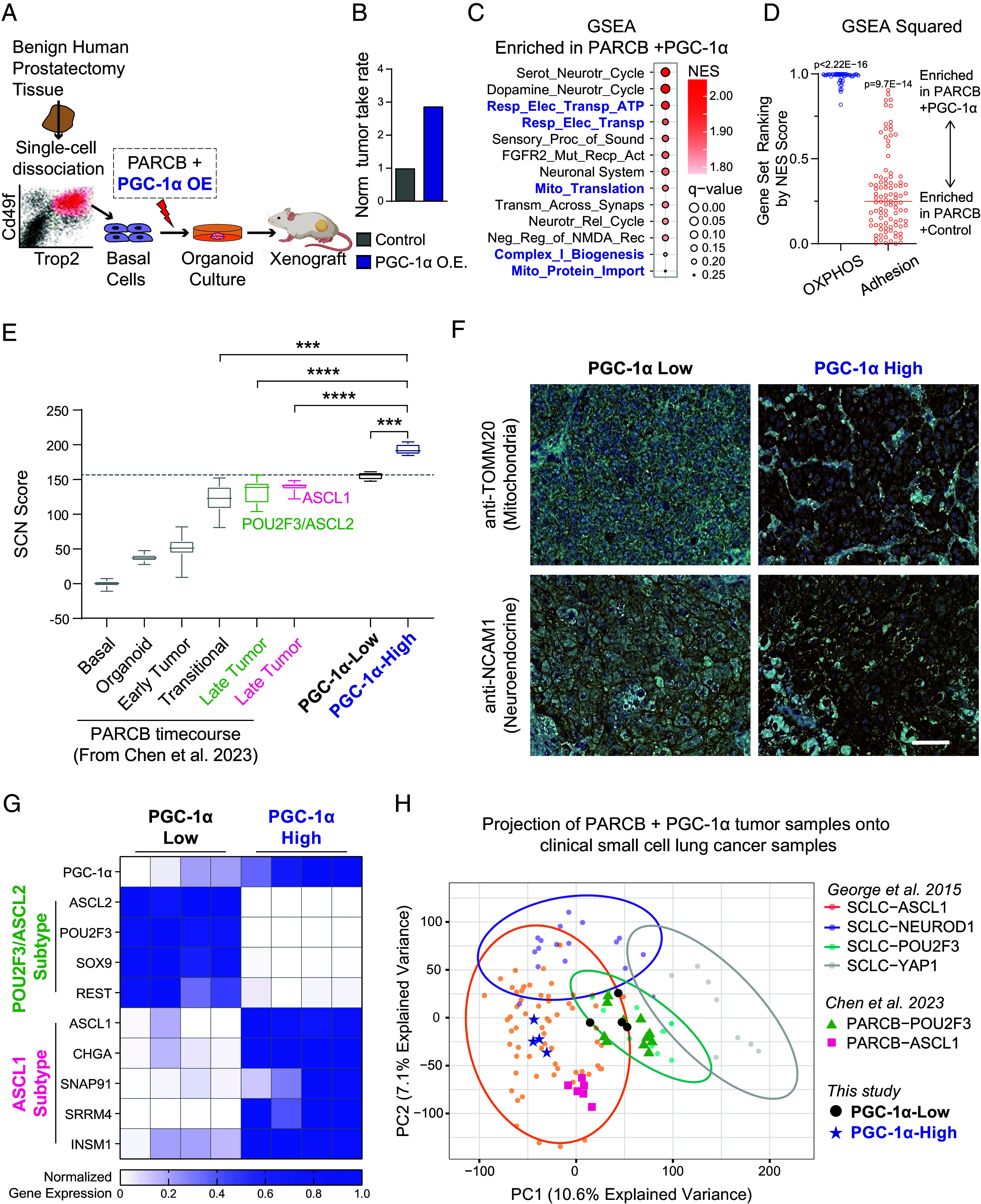
PGC-1α overexpression upregulates OXPHOS and drives SCN prostate cancer toward an ASCL1-expressing lineage. (*A*) Schematic illustrating the experimental setup for assessing the effects of PGC-1α overexpression on PARCB-induced prostate cancer transformation. (*B*) Tumor establishment rate between PARCB transformation using PGC-1α overexpression compared with control. PARCB transformations were performed using three separate patient donors, resulting in a total of 45 xenografts being implanted in 23 mice. (*C*) GSEA in PARCB tumors with PGC-1α overexpression versus control. (*D*) GSEA squared analysis (described in the *Materials and Methods* section and in ref. 1) depicting the distribution of normalized enrichment scores (NES) for thousands of gene sets across multiple gene set families in samples with PGC-1α overexpression versus control. (*E*) A transcriptomic analysis of SCN differentiation comparing SCN scores in PGC-1-High versus PGC-1α-Low PARCB tumors. SCN scores (described in the *Materials and Methods* section and in ref. 1) calculated from datasets derived from Chen et al. (10) are also shown for comparison. (*F*) Immunohistochemistry in histological sections derived from PARCB tumors with high and low levels of PGC-1α. (*G*) A heatmap displaying normalized gene expression levels for signature genes related to POU2F3/ASCL2 and ASCL1 tumor subtypes. (*H*) PCA of PARCB samples, distinguishing between PGC-1α-High and PGC-1α-Low PARCB tumors from this study, overlaid on PARCB tumors from our prior time course study (10) and SCLC cell lines from the CCLE and SCLC patient tumors. Related to *SI Appendix*, Fig. S12. See *SI Appendix* for statistical analyses and datasets used.

We next asked whether PGC-1α overexpression could promote SCN progression during PARCB transformation when the PARCB oncogenes are actively restructuring the chromatin landscape to alter global gene expression. For this, we performed PARCB prostate transformation with PGC-1α overexpression or control (Fig. 6*A*). PGC-1α overexpression resulted in an approximate 100-fold increase in PGC-1α mRNA levels in PARCB prostate organoids (*SI Appendix*, Fig. S12*A*) and a threefold higher tumor take rate compared with controls (Fig. 6*B*). The majority of PGC-1α overexpressing tumors maintained high BFP expression months after transduction, suggesting forward selection during transformation (*SI Appendix*, Fig. S12*B*). Consistently, PGC-1α protein levels were higher in PGC-1α overexpressing tumors compared with control (*SI Appendix*, Fig. S12*C*). These data indicate that PGC-1α overexpression promotes PARCB-induced SCN prostate tumor formation.

To better understand how PGC-1α overexpression promotes PARCB transformation, we performed bulk RNA sequencing on PARCB tumors with PGC-1α overexpression and control. GSEA showed that gene sets related to OXPHOS were among the most significantly enriched following PGC-1α overexpression (Fig. 6*C*). Next, a detailed GSEA squared analysis (1) (described in the *Materials and Methods* section, and in reference (1)) was performed, ranking OXPHOS-related gene sets by NES across multiple gene set families comprised over 10,000 gene sets (Fig. 6*D*). This analysis showed that all OXPHOS-related gene sets (details in *Materials and Methods* section) were highly and exclusively enriched in PARCB tumors with PGC-1α overexpression compared with control (Fig. 6*D*), indicating a robust increase in OXPHOS gene expression. Additionally, we observed that adhesion-related pathways, typically downregulated as cancers transition to a more SCN phenotype (1), were suppressed in PGC-1α overexpressing tumors (Fig. 6*D*). These findings suggest that PGC-1α overexpression may promote SCN differentiation by upregulating OXPHOS.

To quantify the effect of PGC-1α overexpression on SCN differentiation, we first categorized tumors into PGC-1α-High and PGC-1α-Low groups, based on PGC-1α mRNA levels achieved by overexpression. PGC-1α-high samples exhibited a higher SCN score compared with PGC-1α-low (Fig. 6*E*). Notably, PGC-1α-high samples not only displayed increased SCN differentiation in comparison to the PGC-1α-low samples from this study, but also exhibited higher SCN scores than all 55 samples from our recently published PARCB time course study (10) (Fig. 6*E*). Consistently, immunohistochemistry in PARCB tumor sections showed increased mitochondrial content (TOMM20) and neuroendocrine (NCAM1) protein expression in PGC-1α-High tumors (Fig. 6*F*). These results indicate that high levels of PGC-1α overexpression upregulate OXPHOS expression and promote SCN differentiation.

Building on the established relationship between PGC-1α and ASCL1 described above, we investigated whether PGC-1α overexpression could promote lineage plasticity toward the ASCL1 subtype. Indeed, PGC-1α-high samples predominantly expressed genes associated with the ASCL1 subtype, showing a 14-fold increase in expression compared to PGC-1α-low samples. Further, PGC-1α-low samples exhibited nearly a 100-fold increase in the expression of POU2F3/ASCL2 subtype genes compared to PGC-1α-high samples. (Fig. 6*G*). This pattern suggests PGC-1α can potentially promote differentiation toward the ASCL1 lineage.

To determine whether these subtype-specific expression patterns are observable in a broader clinical context, our datasets from PARCB tumors with PGC-1α overexpression were projected onto datasets from SCLC samples in the CCLE and from the clinical study by George et al. 2015 (Fig. 6*H*). We also included the ASCL1 and POU2F3/ASCL2 PARCB samples from our recently published study (10). PGC-1α-high samples clustered well with SCLC-ASCL1 and PARCB-ASCL1 tumors (HC6), whereas PGC-1α-low samples clustered better with SCLC-POU2F3 and PARCB-POU2F3/ASCL2 tumors (HC5). Collectively, these data suggest that PGC-1α promotes SCNC progression toward an ASCL1-expressing subtype with increased OXPHOS capacity.

## Discussion

### PGC-1α-Induced OXPHOS as a Driver of Lineage Plasticity in SCNCs.

Metabolic reprogramming is recognized as a hallmark of many cancers, yet its direct role in driving cancer progression and lineage determination remains unclear. Our findings reveal that mitochondrial metabolism, driven by PGC-1α, is not only tightly correlated with SCNC subtypes but also actively drives SCNC development and lineage plasticity toward the ASCL1 subtype, despite similar oncogenic stimuli across subtypes. These insights pave the way for further investigations into the metabolic underpinnings of SCNCs, suggesting that targeted manipulation of PGC-1α or OXPHOS could offer additional avenues for controlling SCNC progression and differentiation.

### ASCL1 as an Upstream Regulator of PGC-1α.

PGC-1α is influenced by a remarkably diverse array of signals, including hormonal, nutritional, and physiological stimuli, as well as potentially hundreds of transcription factors (53). Despite its complex regulatory network, PGC-1α decisively shapes specific SCNC phenotypes, effectively overriding the oncogenic signals from the same set of oncogenes shared across these subtypes. This observation raises an important question: What specifically regulates PGC-1α in SCNCs in a subtype-specific manner? Our study identifies ASCL1 as a positive regulator of PGC-1α. While analysis of existing ASCL1 ChIP-Seq datasets identified potential ASCL1 binding sites on PGC-1α promoters and gene body, inconsistencies across these datasets—likely due to variations in experimental conditions and ASCL1 antibodies—render the evidence of direct binding inconclusive. Further research is thus necessary to define the precise interaction between PGC-1α and ASCL1.

### The Role of PGC-1α beyond the ASCL1 Subtype.

Recent work identified a fifth neuroendocrine cancer subtype (48), defined by expression of HNF4A—a nuclear receptor that controls hepatic gene regulation. Our study investigated the relationship between PGC-1α, ASCL1, and HNF4A across multiple datasets. Consistent with previous work showing that HNF4A is activated by PGC-1α (54), we found a strong and positive correlation between PGC-1α and HNF4A across multiple SCNC datasets. Further, although most ASCL1-high samples exhibit low HNF4A expression, supporting the idea that these samples belong to a distinct subtype (48), a subset of PGC-1α- and ASCL1-high samples also exhibit high HNF4A expression. This suggests the potential existence of a mixed or transitional population. Thus, future studies should explore the role of PGC-1α in driving lineage plasticity and its contribution to the emergence of this fifth SCNC subtype.

### The Role of PGC-1α Isoforms in Lineage Plasticity and Development of SCNCs.

PGC-1α exists in multiple isoforms, each exhibiting distinct activities depending on the cell type and environmental conditions (55, 56). While our overexpression studies focused on the canonical isoform of PGC-1α, our knockdown construct was designed to target multiple isoforms, including the canonical form. Future studies should aim to characterize the full landscape of PGC-1α isoforms in SCNCs, which may provide deeper insights into how PGC-1α regulates SCNC development and lineage plasticity.

### Parallels with PGC-1α’s Role in Melanoma.

There are several parallels in SCNCs and melanomas, where PGC-1α’s function has been extensively characterized (39, 40, 57–60). In both cancers, PGC-1α promotes mitochondrial biogenesis and OXPHOS, which are important for tumor survival and energy production in tumor subsets with high PGC-1α (39, 40). Another similarity is PGC-1α’s role in regulating metabolic plasticity. In SCNC, the PGC-1α-high ASCL1 subtype exhibits high metabolic plasticity and some resilience to OXPHOS inhibition. Conversely, the PGC1-low POU2F3/ASCL2 subtype is metabolically rigid and highly sensitive to OXPHOS inhibition. This scenario mirrors that in melanoma, where PGC-1α-low melanomas are also highly vulnerable to OXPHOS inhibition (60). However, melanomas with high PGC-1α expression exhibit greater resilience by shifting their metabolism toward glucose and glutamine utilization when OXPHOS is inhibited (60). Thus, future studies should investigate whether PGC-1α-high SCNC subtypes shift toward alternative energy pathways under OXPHOS inhibition.

### OXPHOS Inhibition in Modern Cancer Therapy.

In our study, OXPHOS inhibition had an antiproliferation effect in multiple SCNCs. These findings have clinical implications given the significant interest in targeting mitochondrial metabolism in cancer (26–29). Several inhibitors have been tested in clinical trials with varying degrees of success. For example, CPI-613, aimed at inhibiting key enzymes of the TCA cycle, showed promising phase I outcomes in pancreatic cancer and acute myeloid leukemia but failed to demonstrate a survival benefit in further studies. Another drug, CB-839, which inhibits glutaminase to prevent glutaminolysis feeding into the TCA cycle, did not show benefits in a phase II trial for non–small cell lung cancer. IACS-010759, a potent mitochondrial complex I inhibitor, demonstrated a partial response and alleviation of cancer-related pain in a patient with advanced castration-resistant prostate cancer (61, 62). However, it generally had narrow therapeutic indices and significant toxicities such as lactic acidosis and neurotoxicity.

These challenges may necessitate a more targeted approach, potentially by combining OXPHOS inhibitors with other inhibitors for enhanced efficacy. For instance, the increasing dependency of prostate cancer cells on OXPHOS following ADT (63, 64) may be a compelling case for intervention. The observed upregulation of PGC-1α and OXPHOS post-ADT (Fig. 1*E*) suggests that ADT may drive prostate cancer cells toward a metabolic reliance on OXPHOS, facilitating progression toward the SCN state. Consequently, dually integrating OXPHOS inhibition with ADT may be a strategic approach to potentially halt the progression to SCN prostate cancer early in the treatment regimen.

### Concluding Remarks.

Our study delineates the metabolic heterogeneity within SCNC subtypes, identifying distinct metabolic profiles and capacities for OXPHOS between the ASCL1 and POU2F3/ASCL2 subtypes. We demonstrate that PGC-1α not only distinguishes these subtypes but also drives metabolic reprogramming, which directly alters the cancer phenotype. These findings open additional avenues for research and hold potential for guiding future therapies.

## Materials and Methods

Below is a summary of key materials and methods. All specific protocols, including bulk and single-cell RNA sequencing, cell transduction, drug treatments, microPET/CT imaging, ChIP-Seq analysis, datasets used for bioinformatics analyses (*SI Appendix*, Table S1), and additional technical details can be found in *SI Appendix*.

### PARCB + PGC-1α Transformation.

Donor prostate tissues were transformed using a modified PARCB assay. Basal cells were isolated, transduced with PARCB and PGC-1α lentiviruses, and cultured in Matrigel. Transduced organoids were implanted into NSG mice to initiate tumor formation.

### Cell Lines, Lentiviral Vectors, and Virus Production.

SCN prostate and lung cancer cell lines were cultured in specific media, and routinely tested for Mycoplasma. Custom vectors were designed for PGC-1α manipulation. High-titer lentiviruses were produced for PARCB transformation and transduction experiments.

## Supplementary Material

Appendix 01 (PDF)

## Data Availability

Bulk RNA-sequencing for PARCB + PGC-1a overexpression data have been deposited at dbGAP (phs003230) (65). Bulk RNA-sequencing for PRNBSAN have been deposited at GEO (66).
