# Supplementary material for: PGC-1α drives small cell neuroendocrine cancer progression toward an ASCL1-expressing subtype with increased mitochondrial capacity
Source: Proc Natl Acad Sci U S A. 2024 Nov 26;121(49):e2416882121. doi: 10.1073/pnas.2416882121 (PMC11626175; doi:10.1073/pnas.2416882121)
Supplement: Supplementary file 1 — Appendix 01 (PDF) [file pnas.2416882121.sapp.pdf]

PNAS

Supporting Information for

PGC-1α drives small cell neuroendocrine cancer progression towards an ASCL1-expressing subtype with increased mitochondrial capacity

Grigor Varuzhanyan[1,**], Chia-Chun Chen[2], Jack Freeland[2,3], Tian He[2], Wendy Tran[1], Kai Song[4], Liang Wang[1], Donghui Cheng[5], Shili Xu[2,7,12], Gabriella A. Dibernardo[5,10], Favour N Esedebe[6], Vipul Bhatia[15,16], Mingqi Han[14], Evan R. Abt[2], Jung Wook Park[8], Sanaz Memarzadeh[9,10,11,12,13], David B. Shackelford[12,14], John K. Lee[15,16], Thomas G. Graeber[2,5,7,12,17,18], Orian S. Shirihai[2,3,19,20], Owen N. Witte[1,2,5,12,13,21,*]

Corresponding Authors:

*Owen N. Witte: owenwitte@mednet.ucla.edu

**Grigor Varuzhanyan: gvaruzhanyan@mednet.ucla.edu

This PDF file includes:

SI Methods

Figures S1 to S12

Legends for Figures S1 to S12

Table S1

SI References

SI METHODS

PARCB + PGC-1α transformation

Donor prostate tissues were obtained in a de-identified manner and were therefore exempt from Institutional Review Board (IRB) approval. The PARCB transformation assay was performed as described previously[6] with modifications to the viral constructs to include PGC-1α gain- and loss-of-function analyses. Briefly, donor prostate tissues were digested overnight and isolated cells were stained to identify the basal cells. FACS sorted basal cells were combined with PARCB oncogene lentiviruses at an MOI of 50 in Matrigel (Cat# 356234, Corning). The mixtures of basal cells, viral supernatant, and Matrigel were plated in 48-well plates as hanging drop cultures until Matrigel solidified into domes. 20,000 basal cells were used per Matrigel dome, in a final volume of 20-30 μl per well. After Matrigel solidification, organoids were cultured in prostate organoid media[78] at 37°C and 5% CO2 for 10-14 days. Transduced organoids were harvested by dissociation of Matrigel with 1mg/mL Dispase (Cat# 17105041, Thermo Fisher Scientific) and washed three times with PBS to remove Dispase. Washed organoids were re-suspended in 10 μl of normal Matrigel and 10 μl Matrigel with high growth factors (Cat# 354248, Corning). The organoid-Matrigel mixtures were implanted subcutaneously in immunodeficient NOD.Cg-Prkdcscid Il2rgtm1Wjl/SzJ (NSG) mice[79] to initiate tumor formation. Tumors were extracted before ulceration or reaching around 1 cm in diameter, whichever came first. NSG mice were obtained from the Jackson Laboratories and housed and bred under the care of the Division of Laboratory Animal Medicine at the University of California, Los Angeles (UCLA). All animal handling and subcutaneous injections were performed following the protocols approved by UCLA's Animal Research Committee. Human prostate tissue samples were included in the study if they met the following criteria: (1) tissue sections were free of cancerous cells, and (2) samples exhibited greater than 70% viability upon sorting. Tissue sections containing cancerous cells were excluded from the study Viability was assessed using trypan blue exclusion.

Cell lines and culture media

The SCN prostate cancer line NCI-H660 (Cat# CRL-5813) was purchased from American Type Culture Collection (ATCC). The PARCB-P3-TP5, PARCB-P2-TP6, and PARCB-P7-TP6 cell lines[1] and PARCB-1, PARCB-11[2] cell lines were generated previously. The SCN Lung Cancer Cell lines CORL-311 (POU2F3 subtype), NCI-H209 (ASCL1 subtype) were purchased from ATCC. LNCaP (Cat# CRL-1740) and C4-2B (Cat# CRL-3315), cell lines were purchased from ATCC. SCN prostate and lung cancer cell lines were maintained in stem cell culture media (SCM): Advanced DMEM/F12 (Gibco CAT# 12634028), Glutamax (Gibco, CAT# 35050061), Pen/Strep, B27 (Gibco CAT# 17504044), 10ng/ml human EGF (Peprotech CAT# 100-47, and 10ng/ml human FGF-basic (Peprotech CAT# 100-18B). LNCaP and C4-2B cell lines were maintained in RPMI medium supplemented with 10% FBS, 100 U/mL penicillin and 100 μg/mL streptomycin, and 4 mmol/L GlutaMAX. All cell lines were routinely tested for Mycoplasma using a MycoAlert™ PLUS Mycoplasma Detection Kit (Cat# LT07-703, Lonza).

Lentiviral vectors and high-titer lentivirus production

The following three vectors for PARCB transformation were described previously: mristoylated AKT1 (FU-myrAKT1-CGW), c-MYC and BCL2 (FU-cMYC-P2A-BCL2-CRW), dominant negative TP53 (R175H) and shRNA targeting of RB1 (FU-shRB1-TP53DN-CYW)[6]. For PGC-1α overexpression, a fourth vector was designed. First, an FUGW backbone was subcloned to replace the EGFP with EBFP2 using Gibson assembly (referred to as FUBW). Next, PGC-1α cDNA from pcDNA4 myc PGC-1 alpha (Addgene #10974) was cloned into the FUBW backbone driven by a ubiquitin promoter. An empty FUBW backbone without PGC-1α cDNA was used as a control. For PGC-1α inhibition during PARCB transformation, shRNA targeting PGC-1α was cloned into the myristoylated AKT1 vector (FU-myrAKT1-CGW), downstream of the H1 promoter. The following PGC-1α targeting sequence was used TATGACAGCTACGAGGAATAT. As a control, a sequence that targets no known mammalian genes was used (CAACAAGATGAAGAGCACCAA). For PGC-1α inhibition in cultured cell lines, the same shRNA sequences were cloned into a pLKO_005 backbone, which was subcloned to replace its puromycin

resistance gene along with its promoter with CMV-EBFP2. High titer lentiviruses were made using a previously established method[162].

Transduction of cultured SCN cell lines

Cultured SCN cell lines, which grow in suspension as clusters, were mechanically dissociated using gentle pipetting with a P1000 pipette. 600,000 live cells were seeded in non tissue culture treated 12-well plates and transduced with high titer lentiviruses at an MOI of 2-5 in the presence of polybrene and ROCK inhibitor. The plates were subsequently centrifuged at 1,000 g for 90 min at RT, followed by overnight incubation. The following morning, 1 ml fresh SCM was added to each well and the cells were gently mixed with a P1000 to dislodge any adhered cells. 48-72 hours after transduction, BFP positive cells were sorted to enrich for transduced cells. After a 24-hour recovery period, cells were used for downstream analyses.

PRNBSA-induced SCN prostate cancer transdifferentiation in vitro

PNRBSA transduction experiments were performed as described previously[2]. Cells were seeded in 6-well tissue culture plates at a density of 3 x 105 cells per mL in 3 mL of RPMI medium supplemented with 10% FBS, 100 U/mL penicillin and 100 µg/mL streptomycin, and 4 mmol/L GlutaMAX. Cells were transduced approximately 4-6 hours after seeding at a defined multiplicity of infection (MOI) of 4 for each lentivirus. 72 hours after transduction, cells were trypsinized, washed, and transferred to 100 mm tissue culture plates in 15 mL of neural stem cell media (N-SCM) consisting of Advanced DMEM/F12 medium supplemented with 1X serum-free B27, 10 ng/mL recombinant human bFGF, 10 ng/mL recombinant human EGF, 100 U/mL penicillin and 100 µg/mL streptomycin, and 4 mmol/L GlutaMAX. Media were replenished every 3-4 days. Cells were collected 14 days post-transduction for analysis.

Cell proliferation assay

2,000 - 10,000 cells per cell line in 3-6 replicates were seeded into black, opaque 96-well plates with a glass bottom. Cell content was measured on Days 1-6 using Cell Titer-Glo Luminescent Cell Viability Assay (Cat# G7570, Promega). Luminescence was measured at an integration time of 0.5 second per well. The number of cell doublings were calculated between the day of plating, and the termination of the assay, indicated on each figure panel. All experiments were performed in triplicate to ensure reproducibility. Because CTG uses an ATP-based readout, and since the mitochondrial inhibitors influence the amount of ATP in the cell, the internal GFP, driven by the AKT1 (FU-myrAKT1-CGW) vector was also quantified using microscopy and a plate reader. Consistent results were obtained with both techniques.

Inhibition of PGC-1α and OXPHOS

For pharmacological inhibition of PGC-1α (with SR-18292) and OXPHOS (with IACS-010759 and IMT1B), 96-well plates were pre-filled with SCM media (described above) containing each of the inhibitors or DMSO as a control. Cell growth was quantified using the Cell Titer-Glo Luminescent Cell Viability Assay (Cat# G7570, Promega) as described below.

RT-qPCR

Total RNA was isolated from cells using miRNeasy Mini Kit (Cat# 217004, Qiagen). cDNA was synthesized from 2 ug of total RNA using the SuperScript IV First-Strand Synthesis System (Cat# 18091050, Thermo Fisher). RT-qPCR was performed using SYBR Green PCR Master Mix (Cat# 4309155, Thermo Fisher). Amplification was carried out using the StepOne Real-Time PCR System (Cat# 4376357, Thermo Fisher) and analysis was performed using the StepOne Software v2.3. Relative quantification was determined using the Delta-Delta Ct Method. The following primer pair was used to amplify PGC-1α mRNA:

CCTGCTCGGAGCTTCTCAAA

CCCTTGGGGTCATTTGGTGA

Tissue section, histology, and immunohistochemistry (IHC)

PARCB tumors were fixed by overnight nutation in 10% buffered formaldehyde (SF100-4) at 4℃ followed by three washes in 70% ethanol. Hematoxylin and eosin (H&E) staining was performed by UCLA's Translation Pathology Core Laboratory (TPCL) using standard protocols. TPCL is a CAP/CLIA certified research facility in the UCLA Department of Pathology and Laboratory Medicine and a UCLA Jonsson Comprehensive Cancer Center Shared Facility. For immunohistochemistry, formalin-fixed, paraffin-embedded (FFPE) PARCB tumors were deparaffinized in xylene and rehydrated. Citrate buffer (pH=6.0) was used for antigen retrieval. The sections were incubated in citrate buffer and heated in a pressure cooker. 3% H2O2 in methanol was used to block endogenous peroxidase activity for 10 min at room temperature. The sections were blocked then incubated with primary antibodies overnight at 4°C. Anti-mouse/rabbit secondary antibodies were used to detect proteins of interest and DAB EqV substrate was used to visualize the staining. All components were included in the ImmPRESS Kit (MP-7801-15 and MP-7802-15, Vector Laboratories). The slides were then dehydrated and mounted with Xylene-based drying medium (Cat# 22-050-262, Fisher Scientific).

Western blot

1 million viable cells were lysed on ice using RIPA lysis buffer (Cat#89900, Thermo Fisher). Protein concentrations were measured using the Pierce BCA Protein Assay Kit (Cat#: 23227, Thermo Scientific). Samples were electrophoresed on polyacrylamide gels (Cat# NW04120BOX, Thermo Fisher), transferred to PVDF membranes (Cat# IPVH00010, Millipore). Western blots were visualized using iBright CL1500 Imaging system (Cat#44114, Thermo Fisher).

Antibodies

The following antibodies were used: PGC-1α ms monoclonal 4C1.3 (Sigma, ST1202), TOMM20 ms monoclonal (Santa Cruz Biotechnology, sc-17764), NCAM1/CD56 (Abcam, ab133345), and OXPHOS cocktail ms monoclonal (Abcam, ab110411).

Bulk RNA sequencing and dataset collection

Tumors were dissociated into single cells, followed by cell sorting of quadruple colors (BFP, GFP, YFP, and RFP) by flow cytometry. Total RNA was extracted from the cell lysates using the Zymo Direct-zol RNA Miniprep Plus Kit (Cat. No: R2072). Quality check was performed on the Agilent 4200 Tapestation (Agilent Technology; cat. no. G2991BA). Libraries for RNA-Seq of PARCB with PGC-1α overexpression and control samples were prepared with KAPA Stranded mRNA-Seq Kit (Cat# KK8420, Roche). The workflow consists of mRNA enrichment and fragmentation. Sequencing was performed on Illumina Hiseq 3000 or NovaSeq 6000 for PE 2x150 run. Data quality check was done on Illumina SAV. Demultiplexing was performed with Illumina Bcl2fastq v2.19.1.403 software. Raw sequencing reads were processed through the UCSC TOIL RNA Sequencing pipeline for quality control, adapter trimming, sequence alignment, and expression quantification. Briefly, sequence adapters were trimmed using CutAdapt v1.9, sequences were then aligned to human reference genome GRCh38 using STAR v2.4.2a and gene expression quantification was performed using RSEM v1.2.25 with transcript annotations from GENCODE v23[83].

The FASTQ files of the Park dataset[6], Beltran dataset[33], George dataset[32], Rajan dataset[124] (GSE48403), and Chen dataset were all processed through the TOIL pipeline with the same parameters to get RSEM expected counts. The TOIL-RSEM expected counts of TCGA pan cancer samples were obtained directly from UCSC Xena browser (https://xenabrowser.net/datapages) and RSEM read counts of pan-cancer cell lines from the Cancer Cell Line Encyclopedia (CCLE) were downloaded from DepMap Portal (DepMap Public 22Q1) (https://depmap.org/portal/download/all/). The RSEM counts of all combined datasets were upper quartile normalized with a pseudocount of 1 and log2 transformed (referred to as log2 (UQN+1) counts) and filtered down to HUGO protein coding genes (http://www.genenames.org/) for the downstream analyses. SCLC subtypes[43] and CRPC subtypes[54] were previously defined.

Sequencing of high-grade serous ovarian carcinomas (HGSOC) was approved through UCLA Institutional Review Board (IRB) approved protocols (IRB #10-000727, IRB #20-001626). Tumor

specimens were obtained from high-grade serous ovarian cancer patients who had given their informed consent. RNA was isolated from both solid tumors collected from primary and metastatic sites, and effusion samples such as ascites and pleural fluid. Dissociated or cryopreserved tumor fragments were used for isolation of RNA. These samples were collected at various disease time points: chemonaive, post adjuvant or neoadjuvant chemotherapy, and at disease recurrence. Presence of tumor cells was confirmed on histologic sections. RNA isolation, library preparation, and sequencing were conducted by the Technology Center for Genomics and Bioinformatics (TCGB) core facility at UCLA.

Differential gene expression analysis and hierarchical clustering

Differential expression analysis was performed on raw RSEM expected count data of protein-coding genes using the R package DESeq2[3].

Gene set enrichment analysis (GSEA) and GSEA-squared

Differential gene expression analysis was first performed on raw RSEM expected count data of protein-coding genes on the Rajan 2014[124] dataset between Pre-ADT and Post-ADT samples. Using the fgsea R package (https://github.com/ctlab/fgsea), gene set enrichment analysis (GSEA) was then performed on the hallmark (H), canonical pathways (CP), and gene ontology (GO) gene sets from MSigDB[4]. Additional mitochondria related gene sets were also included from MitoCarta3.0. The ranked list of genes was generated using the signed log2 fold change and -log10 transformed p-values calculated by DESeq2[5]. GSEA results were then ranked by Normalized Enrichment Score (NES) to identify highly enriched gene sets. To investigate pathways broadly related to our highly enriched gene sets (e.g., oxidative phosphorylation and adhesion), GSEA-Squared analysis was performed. In short, gene sets were ranked by NES and marked for if they contained a key term from that category of terms in the pathway name. KS tests were then performed using ks.test.2 to assess the distribution of the category of terms[5]. The keys used were the following:

Oxidative Phosphorylation, OXIDATIVE PHOSPHORYLATION, ELECTRON TRANSPORT, MITOCHONDRIAL COMPLEX, NADH DEHYDROGENASE, MITOCHONDRIAL LARGE RIBOSOMAL; OXPHOS

Adhesion, ADHESION, ADHERENS

Differential gene expression analysis was also performed on raw RSEM expected count data of protein-coding genes on the Chen 2014[32] dataset between HC6/ Class II/ ASCL1+ tumors and the rest of the samples in the dataset. Similar methods were used to perform gene set enrichment analysis on GO BP 2021 gene sets from MSigDB, except genes were ranked by their adjusted p-value.

Principle component analyses

Unsupervised principal component analysis (PCA) was performed on log2 transformed upper quartile normalized RSEM expected count data. The prcomp function in R was run centered and unscaled. Data was projected onto the PCA framework by multiplying the rotation matrix by the projected data's expression matrix.

Small cell neuroendocrine (SCN) score

SCN score was calculated as previously described in Balanis et al[6]. Briefly, PC1 loadings were taken from a pan-cancer PCA which followed non-small cell cancers along their transdifferentiation trajectory towards an SCN phenotype. A higher SCN score indicates a more SCN-like phenotype, while a lower SCN score indicates a more normal, non-SCN-like phenotype.

Motif analysis

ATAC sequencing data was obtained from Chen 2023 dataset[32]. The raw FASTQ files were processed using the published ENCODE ATAC-Seq Pipeline (https://github.com/ENCODE-DCC/atacseq-pipeline). The reads were trimmed and aligned to hg38 using bowtie2. Picard was used to

de-duplicate reads, which were then filtered for high-quality paired reads using SAMtools. All peak calling was performed using MACS3. The optimal irreproducible discovery rate (IDR) thresholded peak output was used for all downstream analyses, with a threshold P value of 0.05. Other ENCODE3 parameters were enforced with the flag-encode3. Reads that mapped to mitochondrial genes or blacklisted regions, as defined by the ENCODE pipeline, were removed. The peak files were merged using bedtools merge to create a consensus set of peaks across all samples, and the number of reads in each peak was determined using bedtools multicov[7]. A variance stabilizing transformation was performed on peak counts using DESeq2[3] and batch effects were removed using removeBatchEffect from limma[8]. DESeq2 was then used to variance stabilize transform read counts and determine hyper- and hypo-accessible peaks across HC5 and HC6 PARCB time course samples, using default parameters and without independent filtering or Cook's cutoff. Peaks were called as hyper or hypo-accessible using abs(log2 fold change)>2 and adjusted p<0.05. Motif analysis was then run separately on hyper- or hypo-accessible peaks in the HC5 versus. HC6 comparison using HOMER[9] with the flags -size 200 and -mask. Motifs were then ranked by their p-value for hyper- or hypo-accessible peak sets. Motifs specific to hyper or hypo accessible peaks were obtained by taking the rank difference of the motifs in the two lists.

Single-cell RNA sequencing analyses

Single-cell expression levels of ASCL1, PGC-1α, ASCL2, and POU2F3 in PARCB time course tumors were extracted from our recently published study[1]. Gene expression was visualized using Uniform Manifold Approximation and Projection (UMAP) analysis as described previously[1]. The downstream quality control, as well as batch integration and correction of PARCB single cell RNA-seq data were performed as described previously[1]. Briefly, visualization of PARCB single cell RNA-seq data was performed using the Seurat (5.0.3) R package[10]. The top 30 principal components were used to perform UMAP analysis. Cell clustering was performed with FindNeighbors function with top 30 principal components and FindClusters function with resolution of 0.5.

Seahorse respirometry

Respirometry experiments were performed on a Seahorse XF96 Extracellular Flux Analyzer (Agilent Technologies). PARCB tumor-derived cell lines were process and counted as described, washed into Seahorse Assay medium (Seahorse XF Base Medium supplemented with 2 mM L-glutamine, 1 mM pyruvate and 10 mM glucose) and immediately seeded into an XF96 microplate, pre-coated with PDL. Cells were plated at a density of 40,000 cells per well in a final volume of 175 µl. Prior to the start of the assay, the XF96 plate was placed in a 37 °C incubator without CO2 for 30 min. During the assay, the following compounds were injected: the mitochondrial ATP synthase inhibitor oligomycin (final concentration 2 µM); the mitochondrial uncoupler FCCP (final concentration 1 µM); and the complex I and III inhibitors rotenone (final concentration 2 µM); and the complex III inhibitor antimycin A (final concentration 2 µM). At the conclusion of the assay, the cells were fixed with 4% paraformaldehyde, stained with Hoechst, and cell number per well was determine based on nuclei number using an Operetta High-Content Imaging System (PerkinElmer). OCRs were normalized to cell number per well.

microPET/CT imaging of tumor metabolism

Tumor-bearing mice were injected with 60 µCi of [^{18}F]-FDG (PETNET Solutions) or [^{18}F]-BnTP (prepared as previously described[11] through tail vein intravenous injections. Following a 60-minute unconscious uptake of the imaging tracer, mice were anesthetized with 2% vaporized isoflurane, and microPET (energy window 350-650 keV, 10-min static scan) and microCT (voltage 80 kVp, current 150 µA, 720 projections, 200µm resolution, scan time 1 min) images were acquired on a GNEXT PET/CT scanner (Sofie Biosciences, Dulles, VA). The microPET images were reconstructed using a 3D-Ordered Subset Expectation Maximization (OSEM) algorithm (24 subsets and 3 iterations), with random, attenuation, and decay correction. The microCT images were reconstructed using a Modified Feldkamp Algorithm. Amide software was used to analyze co-registered microPET/CT images. Representative maximum-intensity-projection (MIP) images are shown.

Chromatin immunoprocepticiation sequencing (ChIP-Seq)

To analyze the regulatory impact of ASCL1 on PGC-1α, we utilized Chromatin Immunoprecipitation Sequencing (ChIP-Seq) and CUT&RUN data from several studies, including Augustyn et al., 2014 (GSE61197), Borromeo et al., 2016 (GSE69398), Cejas et al., 2021 (GSE156290), and Nouruzi et al., 2022 (GSE183198), which were downloaded directly from the Gene Expression Omnibus (GEO). We converted data from bedGraph and wig formats to bigWig format using the UCSC bedGraphToBigWig and wigToBigWig tools to standardize data representation. To align all datasets to a consistent reference genome, we lifted data initially mapped to hg19 over to hg38 using the UCSC liftOver tool. For the Li et al., 2024 ChIP-Seq dataset[2], we merged bigWig files from replicates at each timepoint using the UCSC bigWigMerge tool, aiming to consolidate data for comprehensive analysis. Visualization of the processed datasets was performed with the Integrative Genomics Viewer (IGV 2.17.1), ensuring uniform background noise levels across samples to facilitate accurate comparison.

Sex as a Biological Variable

All prostate tissue samples and cell lines used in this study were obtained from male donors, as prostate tissue is exclusive to men. For the small cell lung cancer (SCLC) cell lines, similar experimental results were observed in cell lines derived from both male and female donors.

Subject Demographics

For human prostate tissues used in the PARCB transformation assay, investigators were blinded to specific patient demographics, including age and weight.

Randomization

Animals were randomly assigned to experimental and control groups. Cell lines and experimental groups were plated in wells in a random order to minimize potential bias. The

randomization process involved placing cell lines in different wells without a predetermined sequence, ensuring that any systematic error in handling or environmental conditions did not influence the results.

Replication and Statistical Analyses

Each biological replicate consisted of at least three technical replicates, and results were averaged to account for variability. All error bars are presented as mean ± SEM, with statistical significance denoted by **** ($p \leq 0.0001$), *** ($p \leq 0.001$), ** ($p \leq 0.01$), or * ($p \leq 0.05$). For correlation analysis, Pearson correlation was performed in GraphPad Prism and the R^2 and p-values are indicated directly on the graphs. All Log2 values have been upper quartile normalized with a pseudocount of 1 (Log2 UQN+1), unless otherwise noted in the figure legends. The data in Figure 3D represent the DepMap dependency scores: a score of zero signifies no proliferation impact, negative values indicate decreased proliferation, and a score of negative one corresponds to the median effect across all pan-essential genes.

Cell Line Authentication:

SCLC cell lines were authenticated using the ATCC Cell Line Identification Service using short tandem repeat (STR) profiling.

SUPPLEMENTAL FIGURES AND LEGENDS

Figure S1. Related to Figure 1

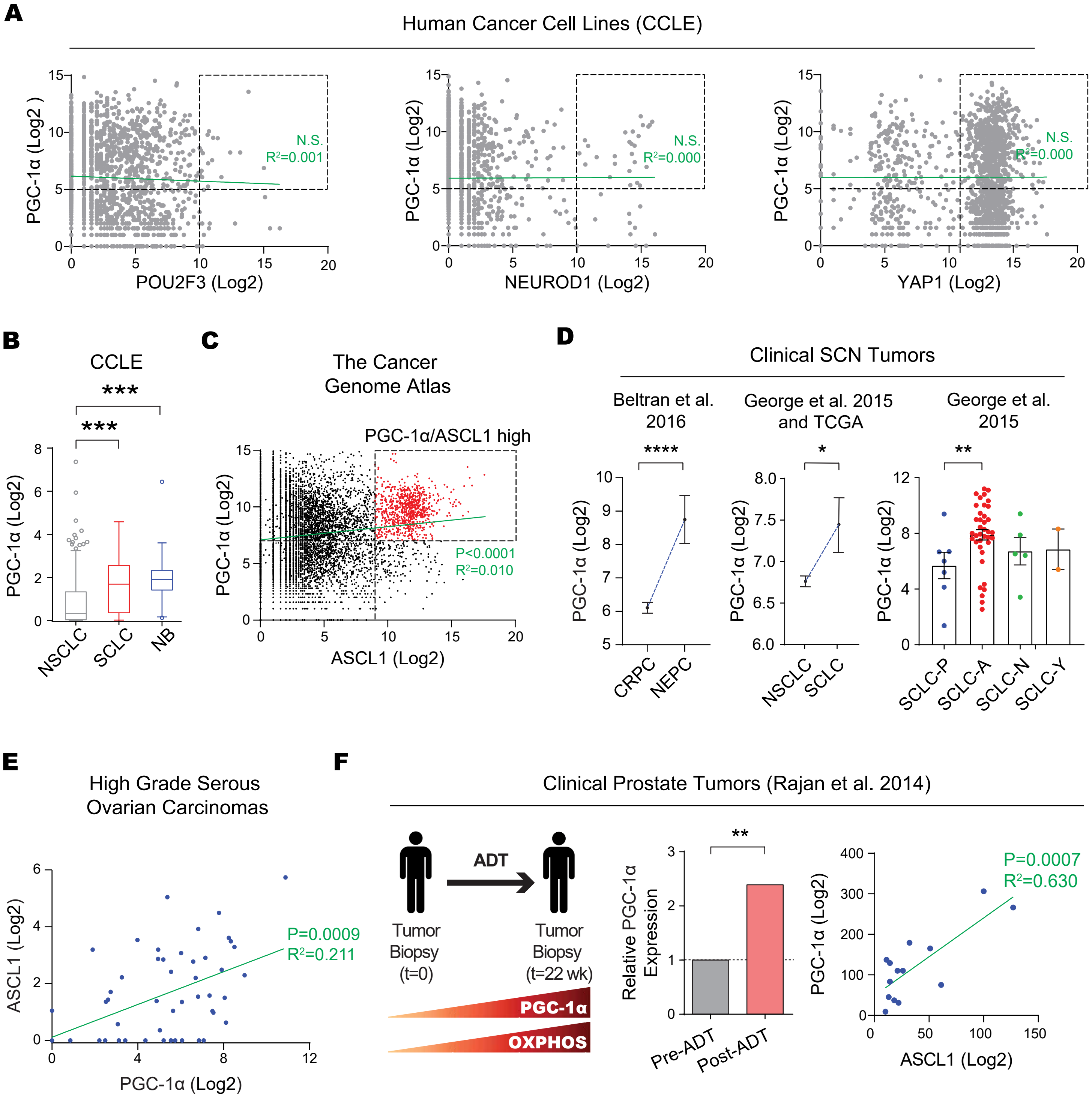

Figure S1. Related to Figure 1.

A. Related to Figure 1A. Gene expression analysis of PGC-1α versus the SCNC lineage markers POU2F3, NEUROD1, and YAP1 in all the human cancer cell lines from the cancer cell line encyclopedia (CCLE).

B. Related to Figure 1A. PGC-1α expression levels in non-small cell lung cancer (NSCLC), small cell lung cancer (SCLC), and neuroblastoma (NB) cell lines from the CCLE.

C. Co-expression analysis of PGC-1α and ASCL1 in all tumor samples from The Cancer Genome Atlas (TCGA).

D. PGC-1α expression levels in multiple SCNC datasets. SCLC subtypes are denoted as follows: P, POU2F3; A, ASCL1; N, NEUROD1; Y, YAP1. The datasets used are indicated.

E. Co-expression analysis of PGC-1α and ASCL1 in a cohort of patients with high-grade serous ovarian carcinomas (HGSOC).

F. Transcriptomic analyses in clinical prostate cancer tumors before and after androgen deprivation therapy (ADT) with enzalutamide. Left panel: a schematic overview. Middle panel: Comparison of PGC-1α expression levels pre- and post-ADT. Right panel: Co-expression analysis of PGC-1α and ASCL1 in (combined pre- and post-treatment analysis). See also Figure 1E.

See the Supplemental Index (SI) Methods for statistical analyses and datasets used.

Figure S2. Related to Figure 2

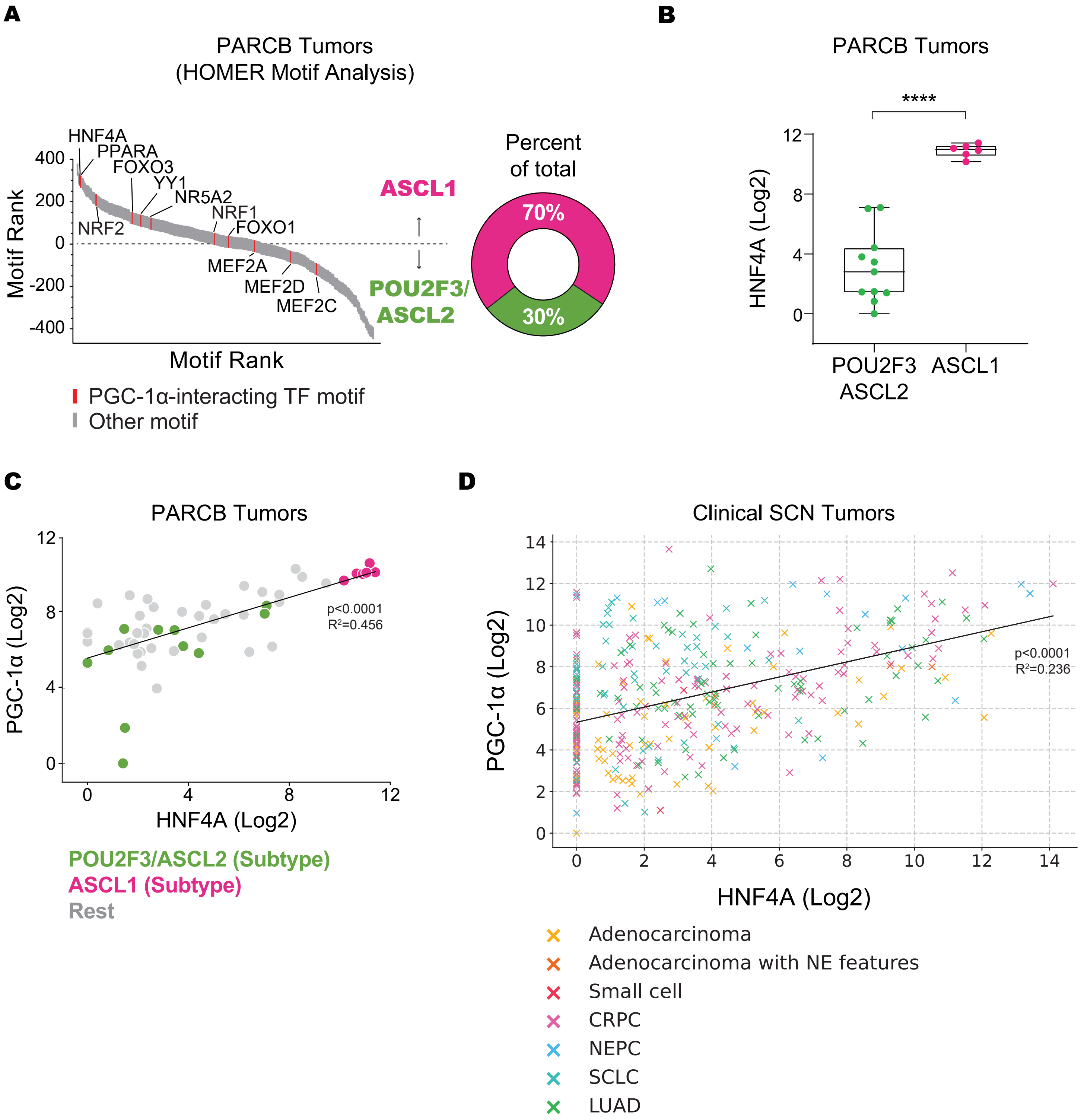

Figure S2. Related to Figure 2.

A. Hypergeometric Optimization of Motif EnRichment (HOMER) analysis across PARCB tumors from both POU2F3/ASCL2 and ASCL1 subtypes. PGC-1α interacting transcription factors were identified from the STRING database[12].

B. Expression levels of HNF4A in PARBC tumors of the indicated subtype.

C. Co-expression analysis of PGC-1α and HNF4A across all PARCB time course samples.

D. Co-expression analysis of PGC-1α and HNF4A across multiple clinical SCNC datasets including lung adenocarcinoma (LUAD), castration-resistant prostate cancer (CRPC), SCN prostate cancer (NEPC), and small cell lung cancer (SCLC)[13–16].

See the SI Methods for statistical analyses and datasets used.

Figure S3. Related to Figure 2

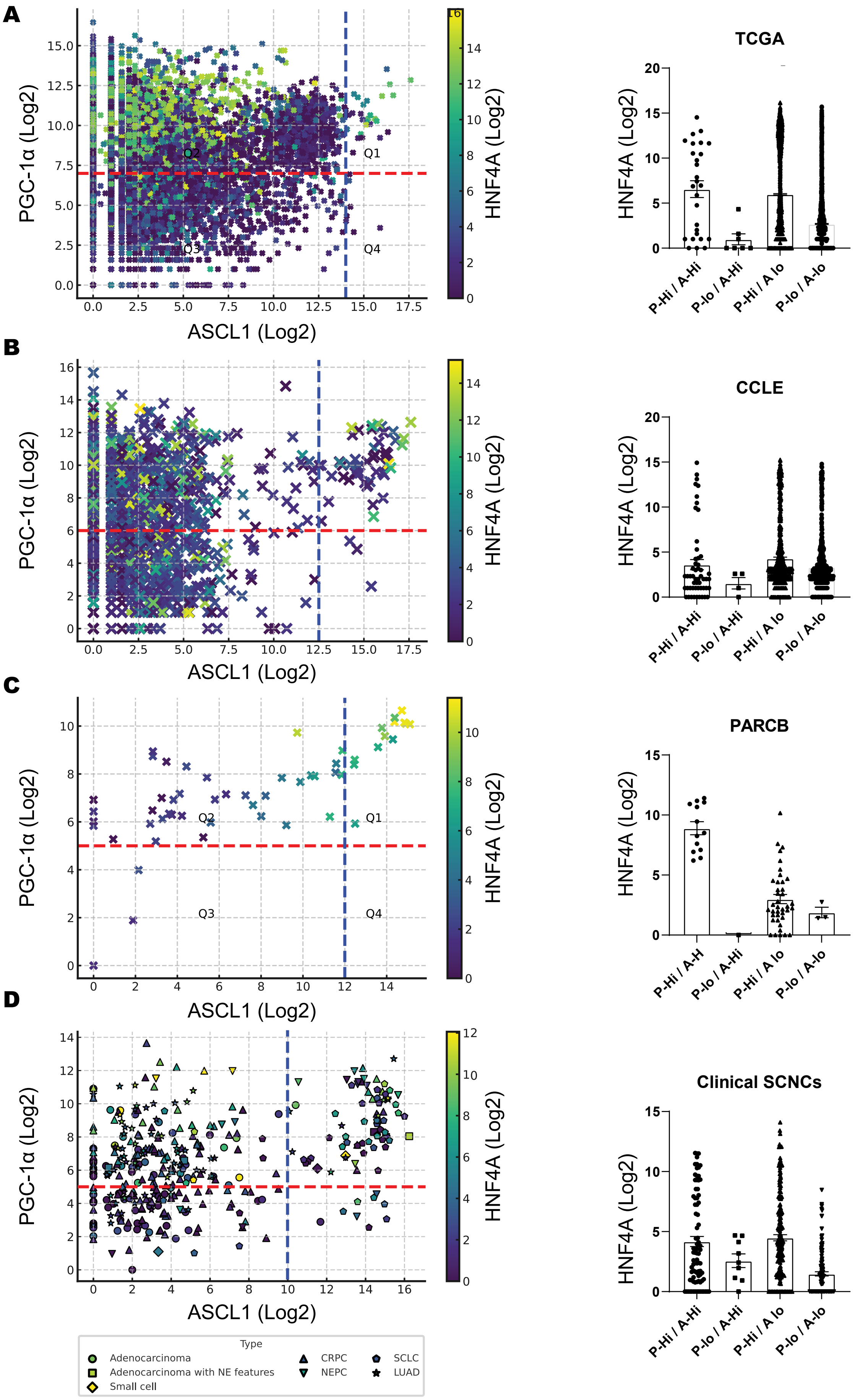

Figure S3. Related to Figure 2.

A. Co-expression analysis of PGC-1α, ASCL1, and HNF4 in all tumors in the TCGA database[17].

B. Co-expression analysis of PGC-1α, ASCL1, and HNF4 in all human cancer cell lines from the CCLE database[18].

C. Co-expression analysis of PGC-1α, ASCL1, and HNF4 in PARCB prostate samples[1].

D. Co-expression analysis of PGC-1α, ASCL1, and HNF4 across multiple clinical SCNC datasets including lung adenocarcinoma (LUAD), castration-resistant prostate cancer (CRPC), SCN prostate cancer (NEPC), and small cell lung cancer (SCLC)[13–16].

See the SI Methods for statistical analyses and datasets used.

Figure S4. Related to Figure 2

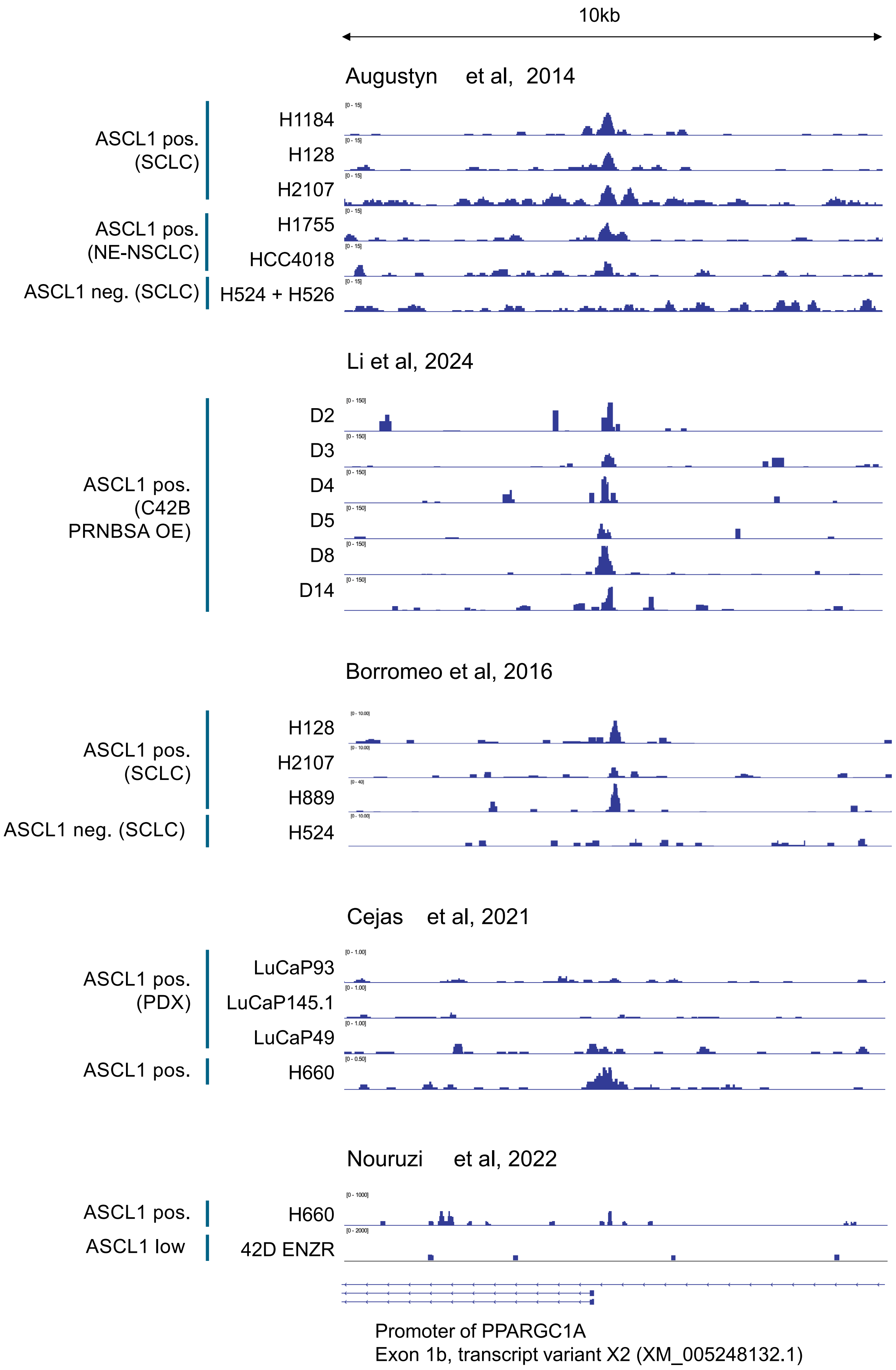

Figure S4. Related to Figure 2

ASCL1 ChiP-Seq analyses showing the alternative promoter of PGC-1α corresponding to variant X2 (XM_005248132.1) encoded by exon1b. The following datasets were used: SCN lung cancer cell lines[19,20], SCN prostate cancer cell lines[2,21], and LuCaP prostate cancer patient-derived xenografts (PDXs)[22].

See the SI Methods for statistical analyses and datasets used.

Figure S5. Related to Figure 2

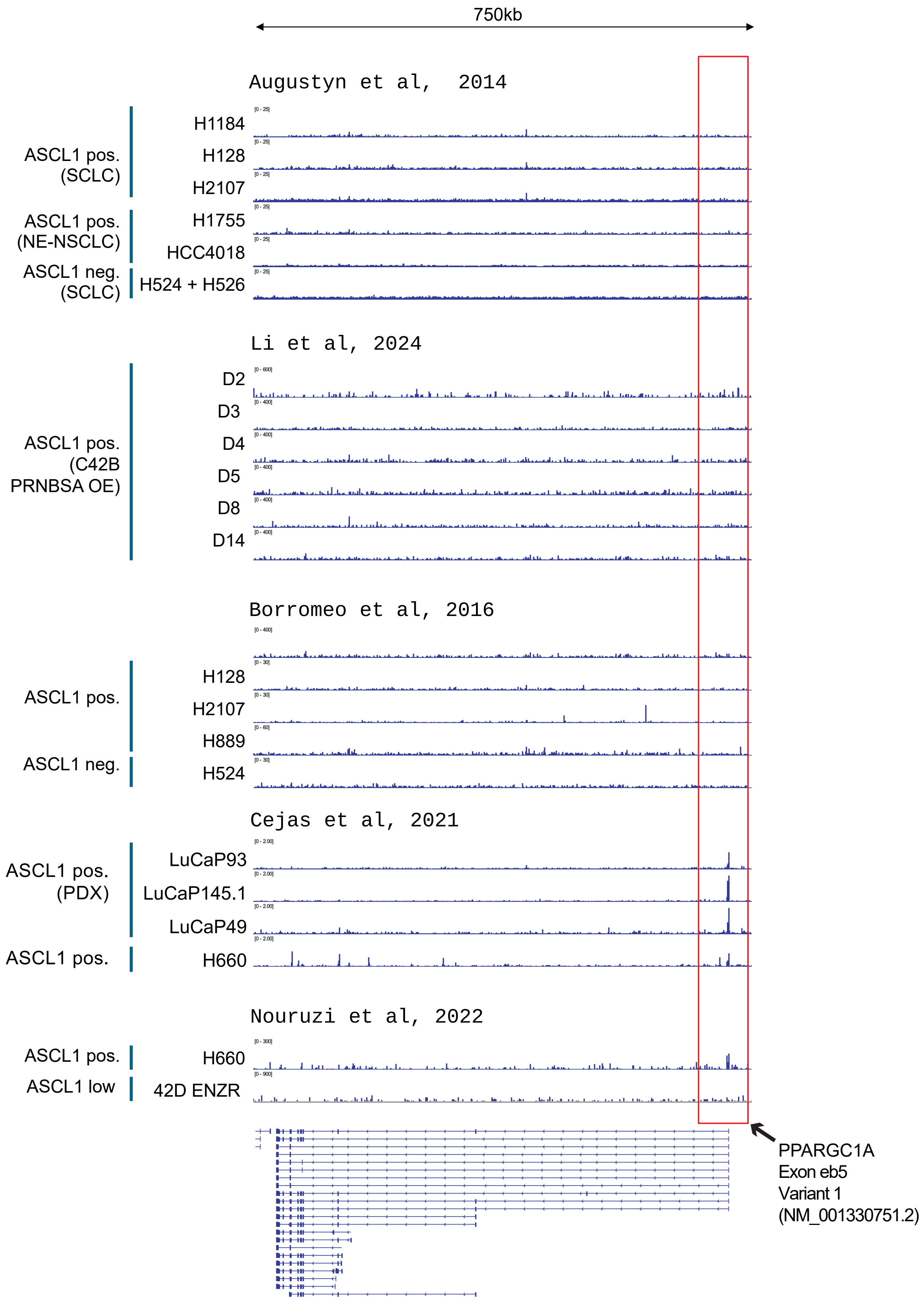

Figure S5. Related to Figure 2

ASCL1 ChiP-Seq analyses highlighting variant 1 of PGC-1α (NM_001330751.2) (red box). The following datasets were used: SCN lung cancer cell lines[19,20], SCN prostate cancer cell lines[2,21], and LuCaP prostate cancer patient-derived xenografts (PDXs)[22].

See the SI Methods for statistical analyses and datasets used.

Figure S6. Rrelated to Figure 2

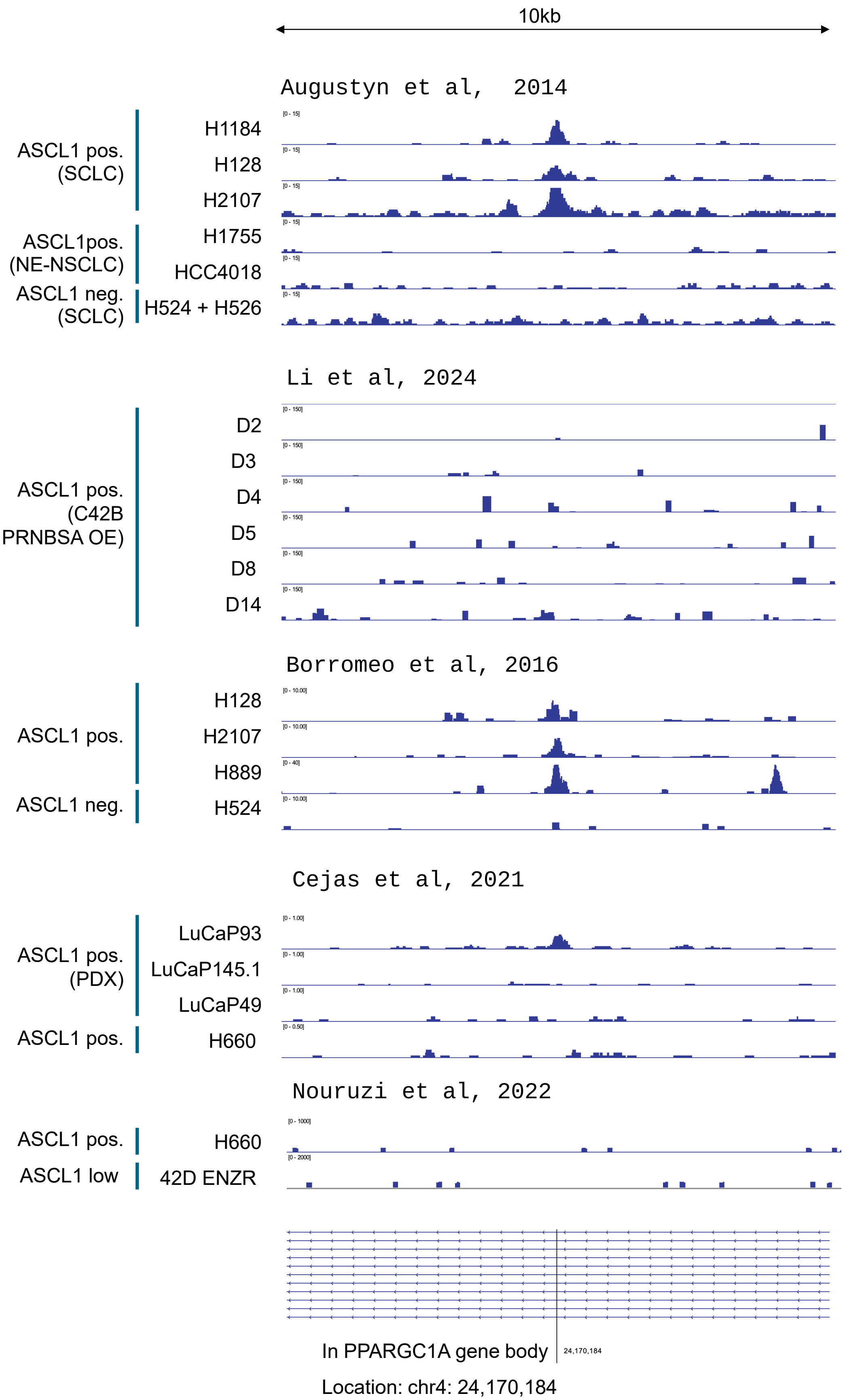

Figure S6. Related to Figure 2

ASCL1 ChiP-Seq analyses indicating a region within the PGC-1α gene body. The following datasets were used: SCN lung cancer cell lines[19,20], SCN prostate cancer cell lines[2,21], and LuCaP prostate cancer patient-derived xenografts (PDXs)[22].

See the SI Methods for statistical analyses and datasets used.

Figure S7. Related to Figure 2

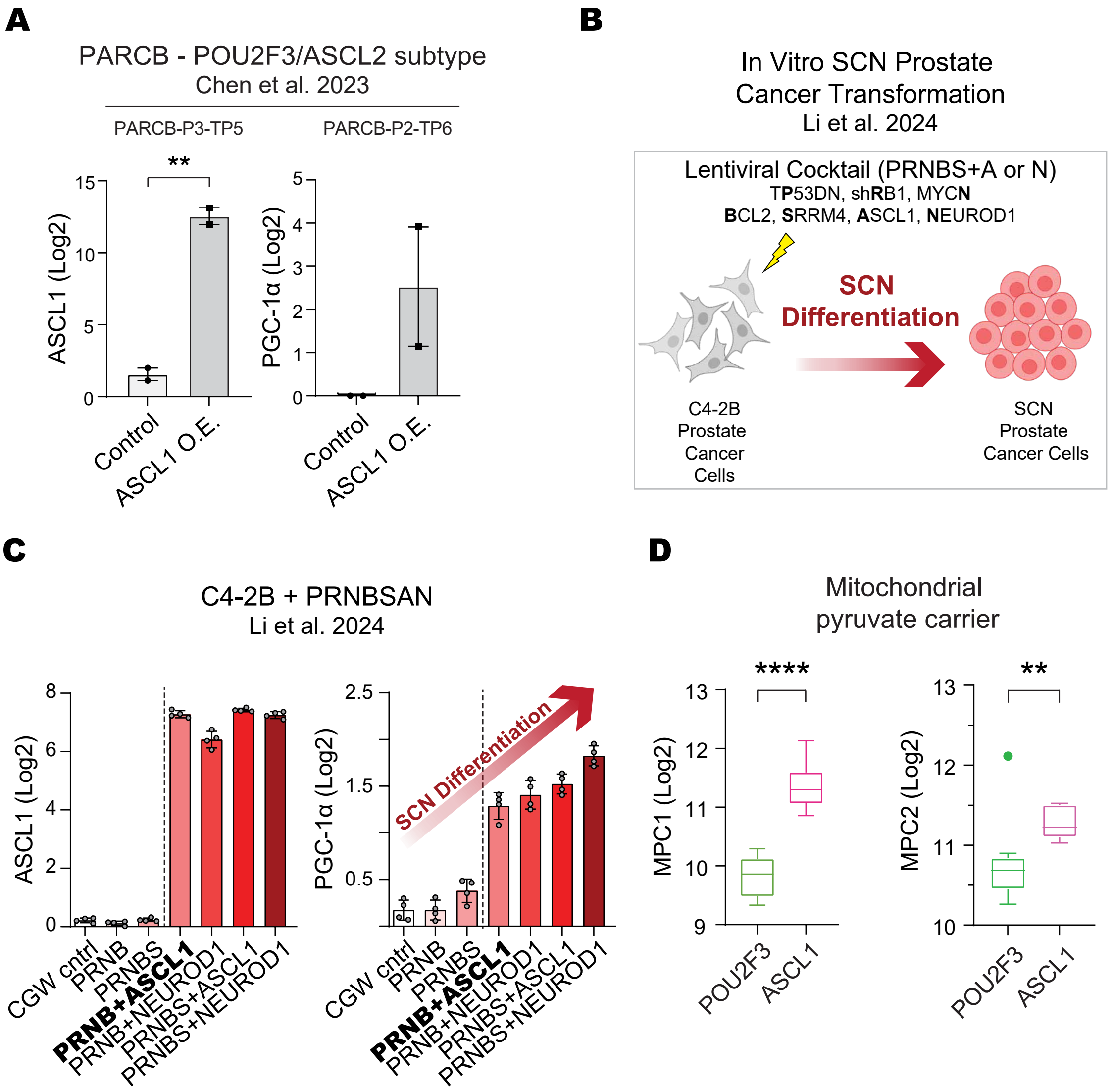

Figure S7. Related to Figure 2

A. Expression levels of ASCL1 (left panel) and PGC-1α (right panel) after acute overexpression of ASCL1 in cell lines derived from the PARCB POU2F3/ASCL2 tumor subtype. Data are mined from Chen et al 2023[1].

B. Schematic illustrating transformation of C42B cells to SCN prostate cancer using the PRNBSAN oncogenes and transcription factors (dominant-negative TP53, shRB1, MYCN, BCL2, SRRM4, ASCL1, and NEUROD1).

C. Expression analysis of ASCL1 (left panel) and PGC-1α (right panel) from C4-2B cells transduced with PRNBSAN. Log2 values are Log2 FPKM+1. The arrow indicates increased SCN differentiation as observed Li et at. 2024[2].

D. Expression of the mitochondrial pyruvate carrier in ASCL1 and POU2F3/ASCL2 PARCB tumor subtypes.

See the SI Methods for statistical analyses and datasets used.

Figure S8. Related to Figure 3

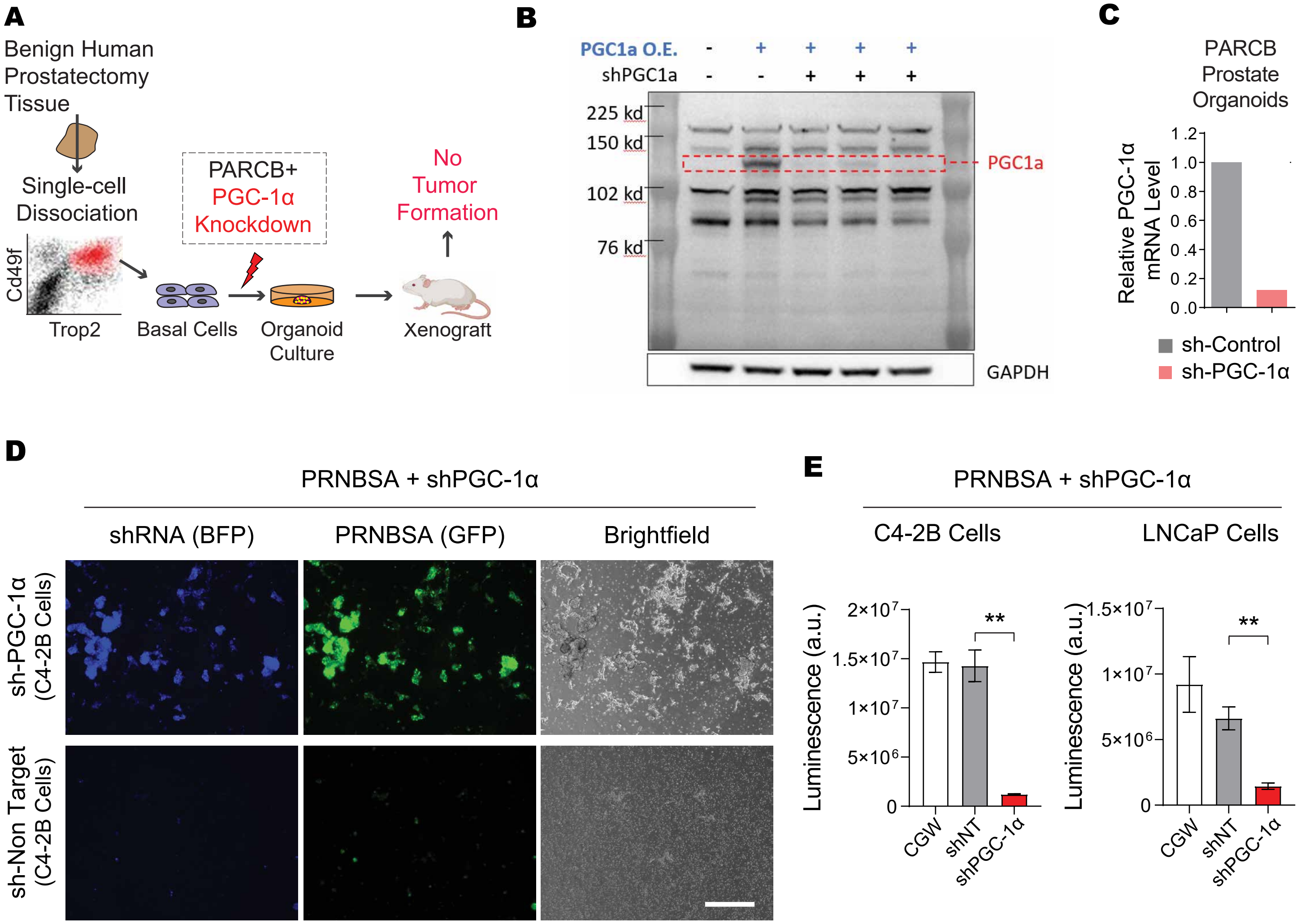

Figure S8. Related to Figure 3

A. Schematic illustrating PARCB prostate transformation with PGC-1α knockdown.

B. PGC-1α protein levels in 293T cells with PGC-1α overexpression and simultaneous inhibition using shRNA.

C. PGC-1α mRNA levels using RT-qPCR in PARCB-transduced prostate organoids.

D. Microscopy analysis of PGC-1α inhibition during PRNBSA-mediated SCN prostate cancer differentiation in vitro. Representative images are shown in C4-2B cells.

E. Cell viability 14 days post transduction PRNBSA and indicated shRNAs. Quantification is shown from both C4-2B (left panel) and LNCaP cells (right panel).

See the SI Methods for statistical analyses and datasets used.

Figure S9. Related to Figure 4

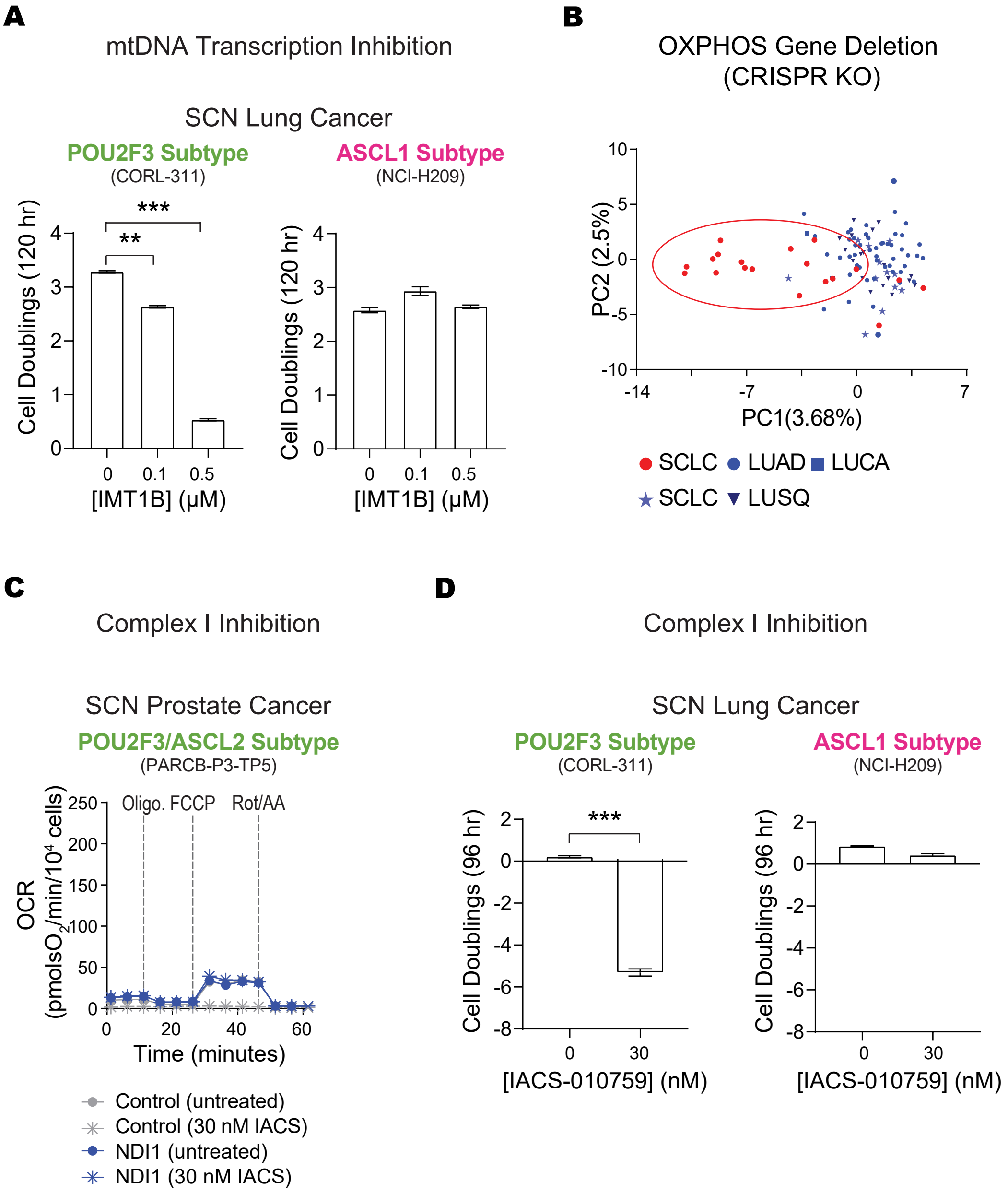

Figure S9. Related to Figure 4

A. Cell proliferation analysis of cell lines derived from the SCN lung cancer POU2F3 and ASCL1 tumor subtypes treated with IMT1B, a mitochondrial DNA-directed RNA polymerase (POLRMT) inhibitor to block OXPHOS.
B. Analysis of differentially dependent genes in NSCLC and SCLC cell lines. Unsupervised PCA on the genetic dependency data from all of the lung cancer cell lines in the DepMap dataset. See SI for details about DepMap score.
C. Seahorse respirometry in cell lines derived from the PARCB POU2F3/ASCL2 tumor subtype with the indicated conditions. See SI for inhibitor details.
D. Cell proliferation analysis of cell lines derived from the SCN lung cancer POU2F3 and ASCL1 subtypes treated with the mitochondrial complex I inhibitor, IACS-010759.

See the SI Methods for statistical analyses and datasets used.

Figure S10. Related to Figure 5

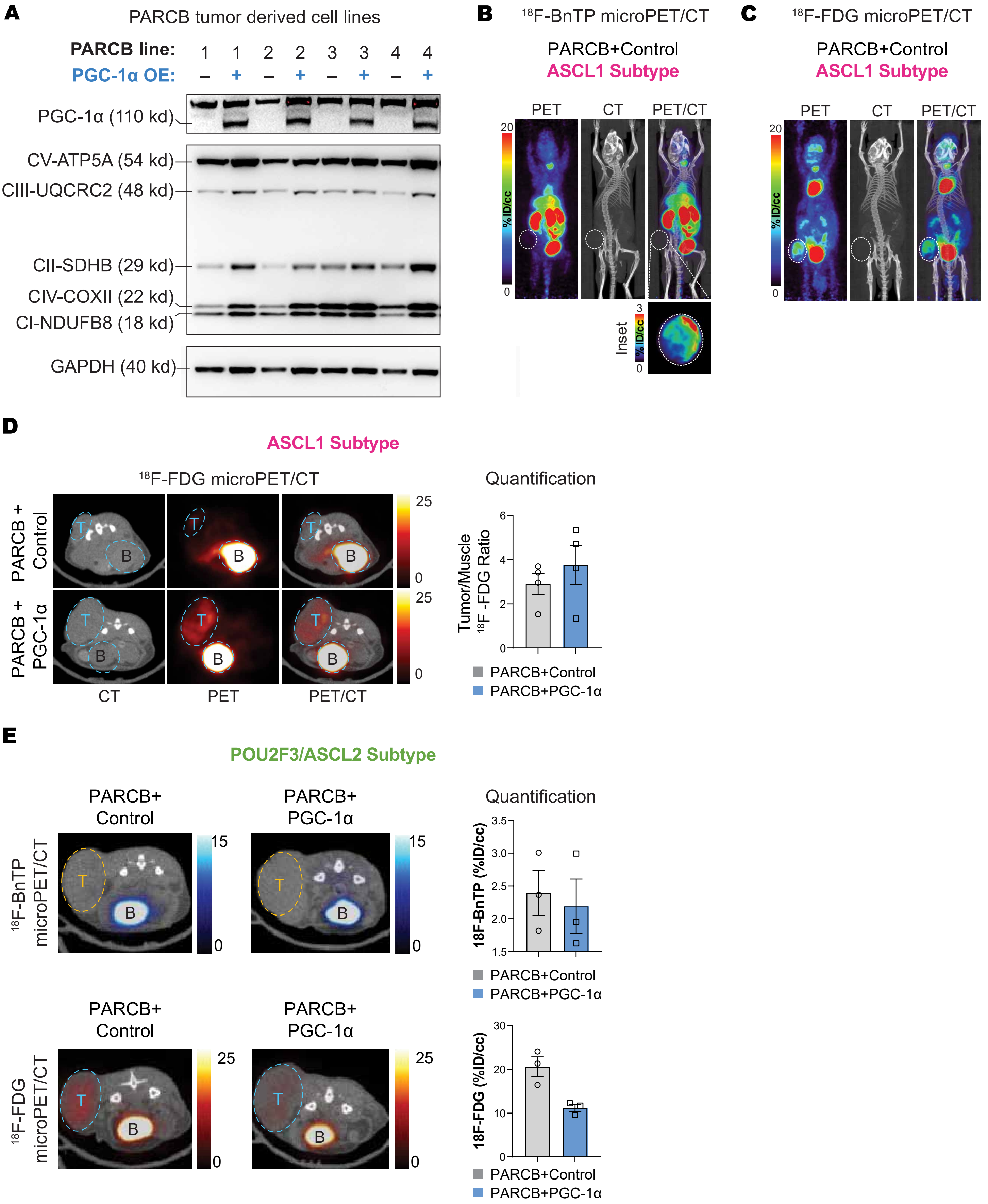

Figure S10. Related to Figure 5

A. Western blot analysis to evaluate the effect of PGC-1α overexpression on the expression levels of all five respiratory chain complexes across a panel of four PARCB tumor-derived cell lines. The PARCB cell lines were generated previously[23]

B. Overlay of in vivo microPET/CT scanning of a mouse with a subcutaneous ASCL1 PARCB tumor imaged with ^{18}F-BnTP indicting mitochondrial membrane potential.

C. Overlay of in vivo micro PET and computed tomography scanning of a mouse with a subcutaneous ASCL1 PARCB tumor imaged with ^{18}F-FDG, indicting glucose uptake.

D. Left panel: Representative ^{18}F-FDG transverse PET-CT images of mice with subcutaneous tumor implantation. Uptake of PET probe was measured as the maximum percentage of injected dose per cubic centimeter (ID%/cc). Tumors are labeled "T", and bladders are labeled "B". Right panel: quantification of ^{18}F-FDG uptake in the indicated groups. Values are normalized to PET signal from adjacent skeletal muscle.

E. Representative ^{18}F-BnTP and ^{18}F-FDG transverse PET-CT images of mice with subcutaneous tumor implantation. Uptake of PET probe was measured as the maximum percentage of injected dose per cubic centimeter (ID%/cc). Tumors are labeled "T", and bladders are labeled "B".

See the SI Methods for statistical analyses and datasets used.

Figure S11. Related to Figure 5

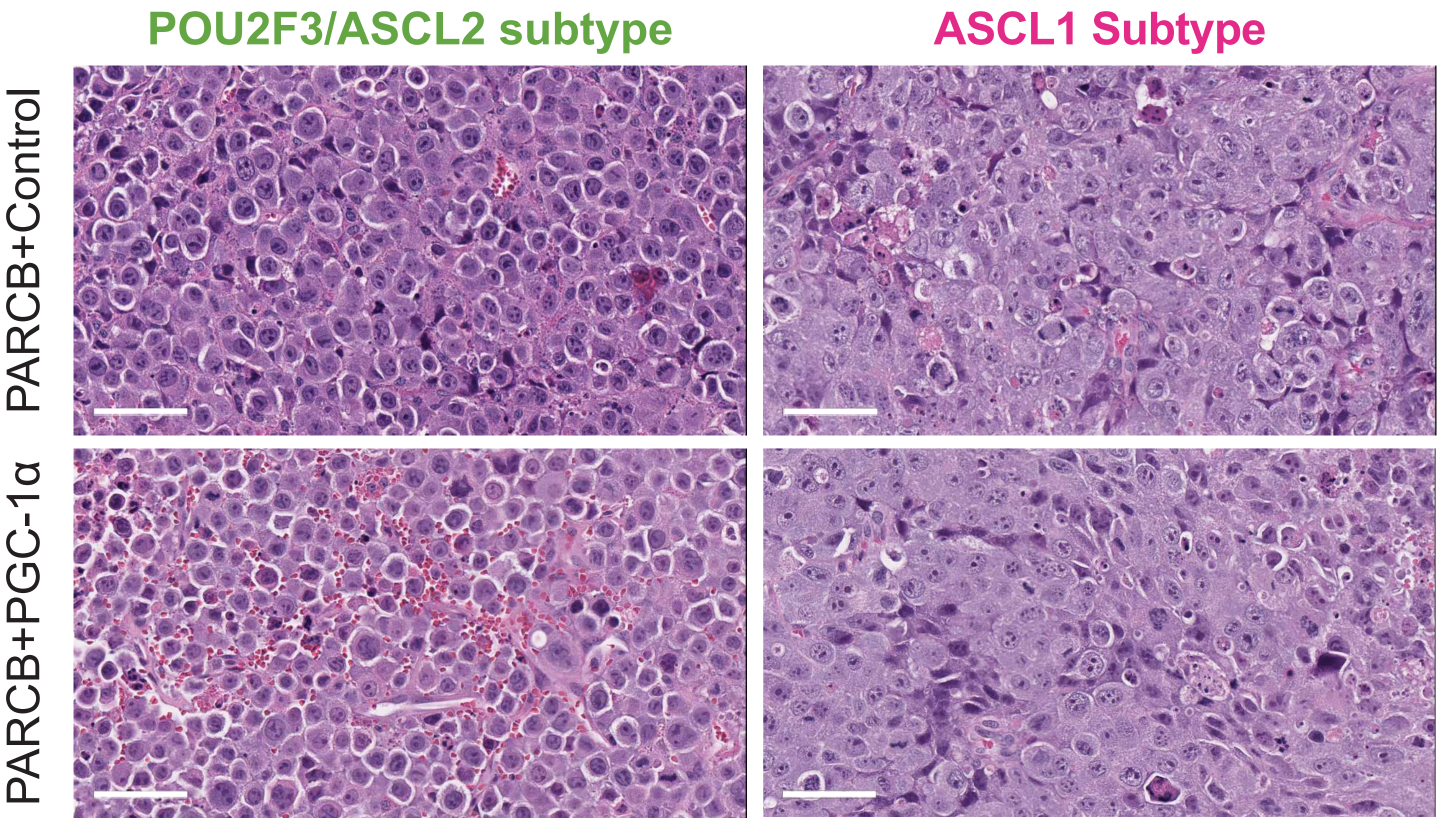

Figure S11. Related to Figure 5

H&E staining in tumor sections from POU2F3/ASCL2 and ASCL1 PARCB tumors with PGC-1α overexpression and control. These tumors were generated from terminally differentiated PARCB cell lines re-injected into mice for tumor growth, as described in Figure 5. Note the increased vasculature of POU2F3/ASCL2 tumors upon PGC-1α overexpression. Scale bars are 60 μm.

Figure S12. Related to Figure 6

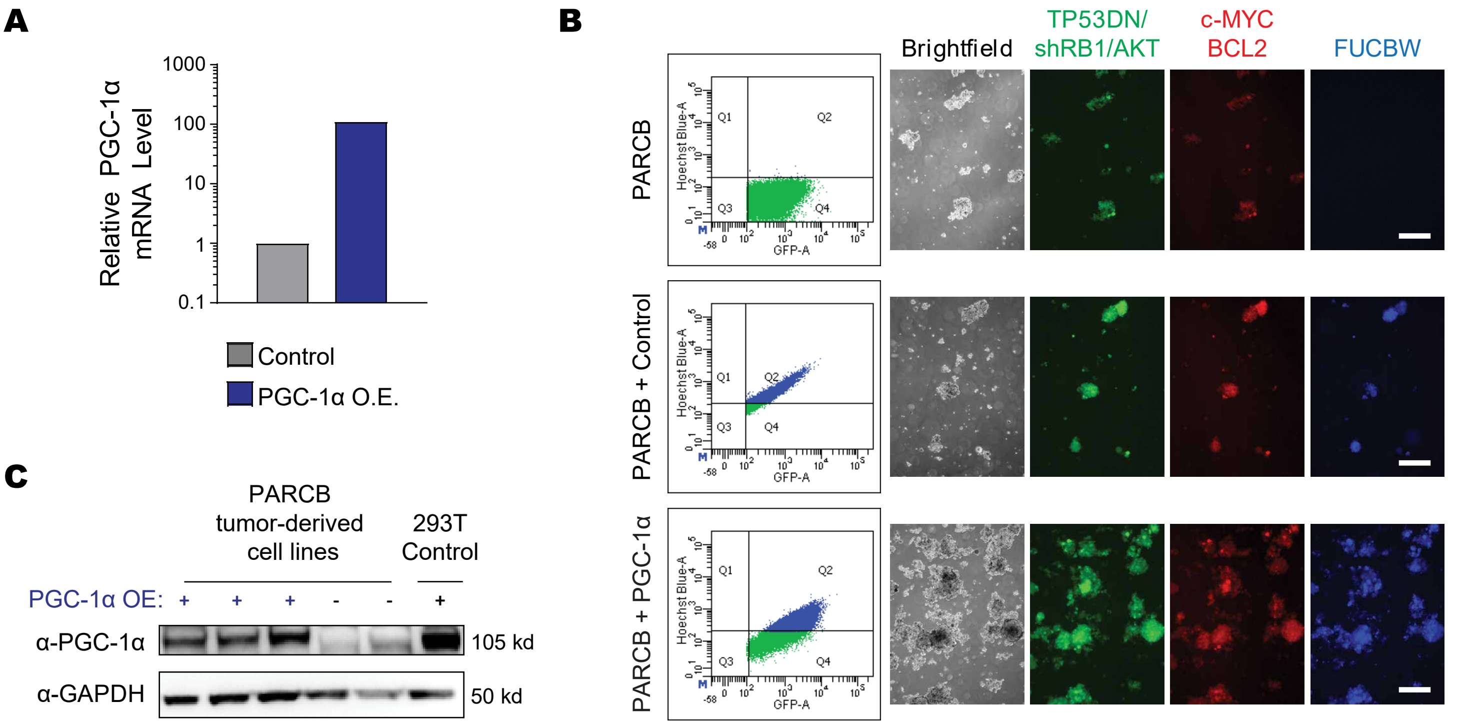

Figure S12. Related to Figure 6

A. PGC-1α mRNA levels using RT-qPCR in PARCB organoids with PGC-1α overexpression versus control. Organoids were harvested just before xenografting.

B. Flow cytometry and fluorescence microscopy analysis of PARCB tumors with PGC-1α overexpression and control. The PGC-1α overexpression and control constructs include a blue fluorescence protein encoded by EBFP2. See SI Methods for more details.

C. Western blot analysis of PGC-1α protein levels in PARCB tumors with PGC-1α overexpression versus control. HEK 293T cells overexpressing PGC-1α were included as an additional control.

Table S1 – Datasets used for Bioinformatics Analyses

**Public Datasets**	Reference
Cancer Cell Line Encyclopedia (CCLE)	18
The Cancer Genome Atlas (TCGA)	17
The Cancer Dependency Map Project (DepMap)	25
**SCNC Model Datasets**	Reference
PARCB	1
PRNBSAN	2
**Clinical Datasets**	Reference
Castration resistant prostate cancer (CRPC) and SCN prostate cancer	13–16
Small cell lung cancer (SCLC)	26
Non-small cell lung cancer (NSCLC)	17
Androgen Deprivation Therapy (ADT)	27
High-grade serous ovarian carcinomas (HGSOC) (unpublished)	N/A
**ChIP-Seq Datasets**	Reference
SCN lung cell lines	19,20
SCN prostate cell lines	2,21
LuCap xenografts	22

SI REFERENCES

1. Chen, C.-C. *et al.* Temporal evolution reveals bifurcated lineages in aggressive neuroendocrine small cell prostate cancer trans-differentiation. *Cancer Cell* **41**, 2066-2082.e9 (2023).
2. Li, S. *et al.* Defined cellular reprogramming of androgen receptor-active prostate cancer to neuroendocrine prostate cancer. *Cell Reports* (In review).
3. Love, M. I., Huber, W. & Anders, S. Moderated estimation of fold change and dispersion for RNA-seq data with DESeq2. *Genome Biol* **15**, 550 (2014).
4. Liberzon, A. *et al.* Molecular signatures database (MSigDB) 3.0. *Bioinformatics* **27**, 1739–1740 (2011).
5. Subramanian, A. *et al.* Gene set enrichment analysis: a knowledge-based approach for interpreting genome-wide expression profiles. *Proc Natl Acad Sci U S A* **102**, 15545–15550 (2005).
6. Balanis, N. G. *et al.* Pan-cancer Convergence to a Small-Cell Neuroendocrine Phenotype that Shares Susceptibilities with Hematological Malignancies. *Cancer Cell* **36**, 17-34.e7 (2019).
7. Quinlan, A. R. & Hall, I. M. BEDTools: a flexible suite of utilities for comparing genomic features. *Bioinformatics* **26**, 841–842 (2010).
8. Ritchie, M. E. *et al.* limma powers differential expression analyses for RNA-sequencing and microarray studies. *Nucleic Acids Res* **43**, e47 (2015).
9. Heinz, S. *et al.* Simple combinations of lineage-determining transcription factors prime cis-regulatory elements required for macrophage and B cell identities. *Mol Cell* **38**, 576–589 (2010).
10. Hao, Y. *et al.* Dictionary learning for integrative, multimodal and scalable single-cell analysis. *Nat Biotechnol* **42**, 293–304 (2024).
11. Han, M. *et al.* Spatial mapping of mitochondrial networks and bioenergetics in lung cancer. *Nature* **615**, 712–719 (2023).
12. Szklarczyk, D. *et al.* The STRING database in 2023: protein-protein association networks and functional enrichment analyses for any sequenced genome of interest. *Nucleic Acids Res* **51**, D638–D646 (2023).

13. Beltran, H. *et al.* Divergent clonal evolution of castration-resistant neuroendocrine prostate cancer. *Nat Med* **22**, 298–305 (2016).

14. Abida, W. *et al.* Genomic correlates of clinical outcome in advanced prostate cancer. *Proceedings of the National Academy of Sciences* **116**, 11428–11436 (2019).

15. Labrecque, M. P. *et al.* Molecular profiling stratifies diverse phenotypes of treatment-refractory metastatic castration-resistant prostate cancer. *J Clin Invest* **129**, 4492–4505 (2019).

16. Beltran, H. *et al.* The Role of Lineage Plasticity in Prostate Cancer Therapy Resistance. *Clinical Cancer Research* **25**, 6916–6924 (2019).

17. The Cancer Genome Atlas Program (TCGA) - NCI. https://www.cancer.gov/ccg/research/genome-sequencing/tcga (2022).

18. Barretina, J. *et al.* The Cancer Cell Line Encyclopedia enables predictive modelling of anticancer drug sensitivity. *Nature* **483**, 603–607 (2012).

19. Augustyn, A. *et al.* ASCL1 is a lineage oncogene providing therapeutic targets for high-grade neuroendocrine lung cancers. *Proceedings of the National Academy of Sciences* **111**, 14788–14793 (2014).

20. Borromeo, M. D. *et al.* ASCL1 and NEUROD1 Reveal Heterogeneity in Pulmonary Neuroendocrine Tumors and Regulate Distinct Genetic Programs. *Cell Rep* **16**, 1259–1272 (2016).

21. Nouruzi, S. *et al.* ASCL1 activates neuronal stem cell-like lineage programming through remodeling of the chromatin landscape in prostate cancer. *Nat Commun* **13**, 2282 (2022).

22. Cejas, P. *et al.* Subtype heterogeneity and epigenetic convergence in neuroendocrine prostate cancer. *Nat Commun* **12**, 5775 (2021).

23. Park, J. W. *et al.* Reprogramming normal human epithelial tissues to a common, lethal neuroendocrine cancer lineage. *Science* **362**, 91–95 (2018).

24. Momcilovic, M. *et al.* In vivo imaging of mitochondrial membrane potential in non-small-cell lung cancer. *Nature* **575**, 380–384 (2019).

25. Tsherniak, A. *et al.* Defining a Cancer Dependency Map. *Cell* **170**, 564-576.e16 (2017).

26. George, J. *et al.* Comprehensive genomic profiles of small cell lung cancer. *Nature* **524**, 47–53 (2015).

27. Rajan, P. *et al.* Next-generation Sequencing of Advanced Prostate Cancer Treated with Androgen-deprivation Therapy. *European Urology* **66**, 32–39 (2014).
